# Inhibition of p21 activates Akt kinase to trigger ROS-induced autophagy and impacts on tumor growth rate

**DOI:** 10.1038/s41419-022-05486-1

**Published:** 2022-12-15

**Authors:** Mayank Maheshwari, Nisha Yadav, Mohammad Hasanain, Praveen Pandey, Rohit Sahai, Kuldeep Choyal, Akhilesh Singh, Mushtaq A. Nengroo, Krishan K. Saini, Deepak Kumar, Kalyan Mitra, Dipak Datta, Jayanta Sarkar

**Affiliations:** 1 Cancer Biology Division, CSIR-Central Drug Research Institute, Lucknow, Uttar Pradesh India; 2 Academy of Scientific and Innovative Research, Ghaziabad, Uttar Pradesh 201002 India; 3 Electron Microscopy Unit, CSIR-Central Drug Research Institute, Lucknow, Uttar Pradesh India

**Keywords:** Macroautophagy, TOR signalling

## Abstract

Owing to its ability to induce cellular senescence, inhibit PCNA, and arrest cell division cycle by negatively regulating CDKs as well as being a primary target of p53, p21 is traditionally considered a tumor suppressor. Nonetheless, several reports in recent years demonstrated its pro-oncogenic activities such as apoptosis inhibition by cytosolic p21, stimulation of cell motility, and promoting assembly of cyclin D-CDK4/6 complex. These opposing effects of p21 on cell proliferation, supported by the observations of its inconsistent expression in human cancers, led to the emergence of the concept of “antagonistic duality” of p21 in cancer progression. Here we demonstrate that p21 negatively regulates basal autophagy at physiological concentration. Akt activation, upon p21 attenuation, driven ROS accumulation appears to be the major underlying mechanism in p21-mediated modulation of autophagy. We also find p21, as a physiological inhibitor of autophagy, to have oncogenic activity during early events of tumor development while its inhibition favors survival and growth of cancer cells in the established tumor. Our data, thereby, reveal the potential role of autophagy in antagonistic functional duality of p21 in cancer.

## Introduction

Rather than merely being considered as a cell cycle regulator, the influence of p21 in several other key cellular programs is cropping up steadily. Over a period of time, a wide range of p21 interacting networks have been explored. Historically, p21 was discovered as a binding partner of CDK2-cyclin E/A complex, later accepted as a universal CDK inhibitor, and in recent years, in addition to cell cycle regulation, it has emerged as a crucial player in diverse cellular processes namely, DNA repair, apoptosis, senescence, transcription, cell migration etc. Being a predominant executor in p53-mediated growth inhibition, p21 is traditionally considered a tumor suppressor. The tumor suppressive effect of p21 is primarily attributed to its inhibitory role for DNA polymerase δ cofactor PCNA [1] and cyclin/CDK complexes [2], slowing cell growth to restore genomic integrity upon genotoxic insults [3] and promoting senescence [4] in response to upstream events. Intriguingly, contrary to its anti-proliferative activity, p21 has also been shown to promote cell survival by a variety of mechanisms including protection from apoptotic cell death, stimulation of cell motility, and supporting assembly of cyclin D-CDK4/6 complex [5]. Thus, the role of p21 in the maintenance of cellular homeostasis is more complex than that was projected initially and it exhibits functional “antagonistic duality”, particularly in regulating cell death mechanisms. Nonetheless, extensive research interests in p21 and other endogenous CDK inhibitors in cancer inspired the idea of synthesizing pharmacological inhibitors for CDKs and this eventually led to the success of palbociclib, a CDK4/6 inhibitor, against breast cancer [6]. Because of its heterogeneous cellular functionality, the concept of targeting p21 for cancer therapy bears the risk of potential side effects. It is, therefore, critical to understand its context-dependent role in tumor progression before exploring further prospects on p21 manipulation in cancer therapy. Being a highly dynamic molecule that plays a key role in multiple cell signaling pathways, it is quite obvious that p21 may also have a direct association with the autophagy pathway.

Autophagy is a cellular housekeeping pathway for the removal of misfolded/aggregated proteins and damaged organelles in order to safeguard replacement with healthy and new ones. This self-degradative process is an important source of energy during development and in nutrient scarcity. Macro-autophagy (hereafter denoted as “autophagy” in this manuscript) is the most comprehensively studied and explored event than two other forms of autophagy i.e. micro-autophagy and chaperon-mediated autophagy. Aberrant regulation of autophagy can lead to several pathological conditions including cancer [7].

Similar to p21, autophagy has contradictory roles in cancer. It can either support cancer cell survival or function as a tumor suppressor depending on the cancer stage, tissue, and context. The tumor suppressive functions of autophagy can be explained by multiple observations namely monoallelic deletion of Beclin-1, a key autophagy regulatory molecule, in sporadic cases of human cancers [8]. It was hypothesized that autophagy depletion causes the accumulation of damaged mitochondria and p62 protein which in turn triggers reactive oxygen species (ROS) [9]. Enhanced free radicals promote DNA damage and chromosomal instability, finally leading to cancer. Additionally, impairment of autophagy in apoptosis-resistant cells induces necrosis in vitro and in vivo [10] leading to the attraction of inflammatory cells and promotes tumor. Further, autophagy has been demonstrated to cause oncogene-induced senescence [11] and thus serving as an early barrier to cancer progression. On the contrary, the catabolic activity of autophagy to recycle energy under nutrient-limiting conditions made them indispensable for cancer cell survival. Because cancer cells have a high demand for nutrients owing to their uncontrolled proliferation and thus they encounter metabolic stress. Direct role of p21 in regulation of autophagy and its underlying mechanism has not been explored yet. Nonetheless, many of the p21-regulated signaling molecules (Ask1, Gadd45, galectin-3, prosaposin etc.) are involved in modulation of autophagy [12–14]. Therefore, it is quite likely that autophagy might be an important factor in addressing “antagonistic duality of p21” in oncogenesis.

In the present study, we investigated the influence of p21 on cellular autophagy and its implication in tumorigenesis. Here, we provide evidence that p21 has a pivotal role in the regulation of autophagy in Akt and ROS-dependent manner. By using genetic and pharmacological perturbation, we found that suppression of p21 is associated with induction of the autophagic process which influences the dynamics of tumor growth in vivo.

## Materials and methods

### Cell culture and treatment conditions

HCT116 p21^−/−^ (JHU cell line# 4) and parental p21^+/+^ (JHU cell line# 36) cells were kind gift from professor Bert Vogelstein (Johns Hopkins University, USA) and were authenticated by STR (short-tandem repeat) profiling. A549, DU145, U2OS, MDA-MB-231, and Detroit 562 cell lines were obtained from American Type Culture Collection. HCT116 and U2OS cells were grown in McCoy’s 5A medium and Detroit 562 cells were maintained DMEM/F-12 medium. Remaining cell lines were cultured in RPMI-1640 medium. Cells were maintained at 37 °C and 5% CO_2_ in respective culture media containing 10% fetal bovine serum (FBS) and 1% antibiotic-antimycotic solution. For p21 suppression, cells were incubated with 10 µM UC2288 (Merck Millipore and Abcam) for 12–24 h.

### Plasmids, cloning, and molecular biology

The plasmid constructs pBABEpuro GFP-LC3 (Ref. No. 22405; created by Dr. Jayanta Debnath), [15] pMRX-IP-GFP-LC3-RFP-LC3ΔG (Ref. No. 84572; created by Dr. Noboru Mizushima) [16] and 1036 pcDNA3-Myr-HA-Akt1 (Ref. No. 9008; created by Dr. William R. Sellers) [17] were obtained from Addgene. Short hairpin RNA sequences specific for non-targeting control, Atg7 [18] and p21 [19] were chemically synthesized from Integrated DNA Technologies, and introduced into pLKO.1 vector via annealed duplex.

For viral packaging, respective recombinant plasmids were transfected into either phoenix ampho (for retroviral vector) or 293 T (along with pSPAX2 and pMD2G packaging plasmids for lentiviral vector) cells by calcium phosphate co-precipitation method. Packaged viral particles were collected by harvesting the supernatant at 48 h and 72 h post-transfection and subsequently used for transient or stable transduction of cells. Stable cell pools were generated by culturing infected cells in presence of respective selection antibiotics at the appropriate concentration.

### Western blotting

At the end of the experimental procedures, cells were lysed in lysis buffer composed of 25 mM HEPES (pH 7.2), 0.4 M NaCl, 1.5 mM MgCl_2_, 0.2 mM EDTA, 1% NP-40 and containing protease and phosphatase inhibitor cocktail (Roche). After determining the protein concentration by BCA protein assay kit (Thermo), 10–30 µg of protein lysates from each sample was resolved in SDS-PAGE and thereafter transferred onto polyvinylidene difluoride membrane. Blots were subjected to blocking for 1 h at RT and probed overnight at 4°C with specific primary antibodies followed by appropriate HRP-linked secondary antibodies (Sigma-Aldrich). After brief incubation of membranes with enhanced chemiluminescence (ECL) solution, protein bands were visualized by Bio-Rad XRS + ChemiDoc Imaging System. Following primary antibodies were used in this study: anti-β actin (A2228, Sigma-Aldrich), anti-GAPDH (ABM22C5, Abgenex), anti-LC3B (L7543, Sigma-Aldrich), anti-Atg7 (8558S, CST), anti-p53 (sc-126, Santa Cruz Biotechnology), anti-p21 (2947S, CST), anti-Akt (4685S, CST), anti-pS473 Akt (4051S, CST), anti-pT308 Akt (2965S, CST), anti-mTOR (2983S, CST), anti-pS2481 mTOR (2974S, CST), anti-pS2448 mTOR (5536S, CST), anti-4E-BP1 (9644S, CST), anti-pT37/46 4E-BP1 (2855S, CST), anti-sestrin (11431-2-AP, Proteintech), anti-catalase (12980S, CST), anti-MnSOD (13141S, CST), anti-FoxO3a (2497S, CST), anti-pS253 FoxO3a (9466S; CST), anti-FoxO1 (2880S, CST), anti-pS256 FoxO1 (9461S, CST), anti-GSK-3β (9315S, CST), anti-pS9 GSK-3β (5558S, CST).

### Confocal microscopy

Cells were plated onto poly-L-lysine coated round coverslips in a 6-well plate, grown overnight and subjected to experimental conditions as indicated. Stable cell lines expressing recombinant fluorescence proteins were imaged immediately after mounting on glass slides with ProLong™ Gold Antifade Mountant with DAPI (Thermo). For immunofluorescence, cells were fixed with 4% paraformaldehyde, briefly washed with PBS, and subsequently permeabilized with 0.5% Triton X-100 in PBS for 10 min followed by 1 h blocking in 2% BSA at RT. Coverslips were then incubated with anti-LC3A/B antibody (4108 S, CST; 1:250) for overnight at 4 °C, washed three times with PBS, and incubated again with DAPI and Alexafluor™ 594 conjugated anti-rabbit secondary antibody (A11012, Thermo; 1:250) for 1 h at RT. After 3 washes with PBS, coverslips were mounted on glass slides with ProLong™ Gold antifade reagent and examined under LSM510 META (Carl Zeiss) and BX61WI (Olympus) confocal microscopes. LC3-specific puncta were counted manually by ImageJ in minimum 25 cells per experimental group.

### Electron microscopy

The study was performed according to the protocol described earlier [18, 20]. Briefly, cells were fixed overnight with 2.5% glutaraldehyde (in 0.1 M phosphate buffer; pH 7.4) at 4 °C and subsequently post-fixed with 1% OsO_4_ followed by encapsulation in agarose. Cells were then embedded in Spurr resin after dehydration with ascending grades of ethanol. After polymerization, ultrathin sections (60–70 nm) were obtained using Leica EM UC7 microtome and subsequently collected onto 200 mesh copper grids. Sections were double-stained with uranyl acetate and lead citrate and examined under JEOL JEM 1400 transmission electron microscope equipped with Gatan Orius 830 CCD camera at 80 kV. Images were analyzed by Gatan DigitalMicrograph software.

### Flow cytometry

For measuring endogenous ROS, cells were stained with CM-H_2_DCFDA (Thermo) fluorescent probe following manufacturer protocol. Briefly, 6 × 10^5^ cells were grown overnight in six-well plate at 80–90% confluency and subsequently incubated with 5 µM CM-H_2_DCFDA at 37 °C for 30 min in dark. After a brief wash with PBS, cells were harvested by trypsinization and resuspended in PBS before being analyzed by flow cytometry (FACS Calibur, Becton Dickinson).

For LC3 degradation assay, overnight grown HCT116 p21^+/+^ cells with stable expression of GFP-LC3 were incubated as indicated. Cells were then trypsinized, washed with PBS, and resuspended in HBSS containing 2% FBS. Analysis of 3 × 10^5^ cells from each sample was carried out by flow cytometer.

### Colony formation assay

In vitro colony formation assay was performed in six-well plate as previously described [21]. Approximately 500 numbers of HCT116 p21wt and null cells were seeded onto each well and allowed to grow in recommended culture media containing 10% FBS with periodic replenishment on every 4th day. After 14 days of incubation, when colonies were visible, cells were fixed after brief wash with PBS. Fixed colonies were subsequently stained with 0.5% crystal violet (in methanol) for 30 min at RT and thereafter rinsed with tap water to remove residual dye and left to dry in normal air at RT before imaging.

For the experiment with UC2288, HCT116 p21wt cells were plated at a density of 1000 cells/well in six-well tissue culture plate. After overnight growth, cells were exposed to 10 μM UC2288 for six days with replenishment of media every 2 days. Colonies were then stained with crystal violet and imaged as described above. Images were analyzed with ImageJ for the area of cell coverage.

### Mice and generation of xenograft tumors

Animal experiment was performed with prior approval of study protocol from the Animal Ethics Committee of the CSIR-Central Drug Research Institute. Five to six weeks old nude or NOD/SCID mice were inoculated subcutaneously with 5 × 10^6^ HCT116 p21wt and null cells (suspended in 100 µl PBS) at right and left flank region respectively of same mouse. The animals were given *ad libitum* access to feed and water and observed regularly to monitor tumor induction. At ~7–10 days post-injection, when tumors were palpable, their size (length and width) was measured using calipers at every 3rd day till 22nd to 25th days post implantation. The tumors were allowed to grow to its maximum size without any sign of morbidity of the experimental animals. Overall health and weight of each experimental mouse was monitored precisely throughout the study. Tumor volume (TV) was calculated by the following equation: *V* (mm^3^) = ½ × *D* × *d*^2^ (*D* = large diameter, *d* = small diameter). At the end of the study (3 weeks post inoculation), animals were sacrificed by CO_2_ asphyxia and tumors were excised surgically for further processing.

### Statistics

Statistical analyses of values between two groups were performed by two-tailed Student’s *t* test using GraphPad Prism software or Microsoft excel. Results of three independent experiments were expressed as mean ± SEM. Results with *P* values < 0.05 were considered significantly different.

## Results

### p21 regulates basal level of autophagy at physiological concentration

**Fig. 1:**
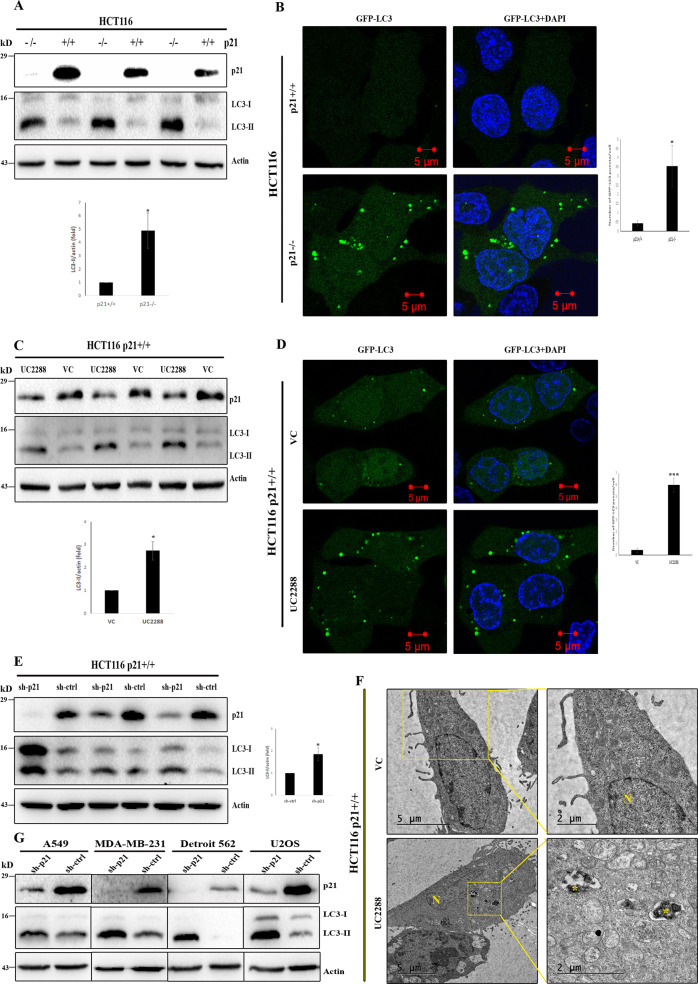
p21 inhibits autophagy. Western blot analysis and densitometric quantitation (*n* = 3; mean ± SE) of LC3 turnover of three biological replicates in HCT116 cells before and after p21 suppression by genetic deletion (**A**), pharmacological inhibition (**C**), and RNA silencing (**E**) (*P < 0.05). **B** Representative images of LC3-specific puncta and their quantification (>25 cells) in HCT116 p21wt and null cells with stable expression of GFP-LC3 (*P < 0.05). **D** Punctate GFP-LC3 fluorescence in HCT116 p21wt cells treated with vehicle and UC2288 (p21 inhibitor) and their quantification from >25 cells (***P < 0.001). **F** Electron micrographs of HCT116 cells that have received no treatment or incubated with UC2288. Distinct autophagic vesicles are visible in magnified photographs after UC2288 treatment. N- nucleus; Asterisk- autophagic vacuoles. **G** Lysates from control and p21 shRNA transduced A549, MDA-MB-231, Detroit 562, and U2OS cells were analyzed by immunoblotting with indicated antibodies to examine the effect of p21 suppression on LC3 turnover across cell lines of different cancer origins.

In the present study, we first explored whether p21 has any role on basal level of autophagy. To this end, HCT116 p21^+/+^ (wt) and p21^−/−^ (null) cells were used to compare lipidation of LC3 which is the signature marker for autophagy induction. Here, HCT116 p21null cells showed a robust increase in (lipidated) LC3-II as compared to the parental p21 wild-type cells (Fig. 1A). Consistent with this observation, visualization of autophagy in GFP-LC3 expressing stable cell lines by confocal microscopy study revealed distinctive punctate fluorescence, indicative of autophagosome accumulation, in p21null cells. In contrast, HCT116 p21wt cells showed diffuse pattern of GFP-LC3 distribution (Fig. 1B). To further confirm involvement of p21 in autophagic process, we used a pharmacological inhibitor of p21 called UC2288 that attenuates p21 activity transcriptionally as well as at post-transcriptional level [22]. Incubation of HCT116 p21wt cells with UC2288 at 10 μM concentration for 24 h caused reduction in p21 expression. This was associated with increased turnover of LC3-II (Fig. 1C). Likewise, treatment of UC2288 to GFP-LC3 stable cell line with p21wt background led to the formation of LC-3 specific puncta (Fig. 1D). The results were further validated by immunostaining of LC3 in HCT116 p21wt cells before and after incubation with UC2288 (Supplementary figure 1A, B). The observations were additionally authenticated by knocking down p21 expression by shRNA-mediated gene silencing in p21wt cells which were associated with marked increase in the conversion of LC3-I to LC3-II (Fig. 1E). In agreement with above results, restoration of p21 expression in HCT116 p21null cells resulted in reduction of LC3-II level (Supplementary figure 1C) and thereby confirming an inhibitory role of p21 in cellular autophagy. Next, we investigated ultrastructural changes associated with p21 inhibition. TEM study of UC2288 treated HCT116 p21wt cells showed accumulation of typical autophagic vacuoles with engulfed cargo while vehicle-treated healthy cells exhibited normal sub-cellular morphological features (Fig. 1F) and thus validating autophagy induction upon p21 suppression. To further examine generality of above results, we suppressed p21 activity by RNAi in different human cancer cell lines including head and neck cancer (Detroit 562), breast cancer (MDA-MB-231), osteosarcoma (U2OS), and lung cancer (A549). Analysis of whole-cell protein lysates by immunoblotting revealed that irrespective of cell type, attenuation of p21 conferred induction of autophagic vacuolization and was evident as increased turnover of LC3-II in p21-depleted cells (Fig. 1G). All of these findings suggested that p21 is a physiological regulator of autophagy.

### Mechanism of autophagic vacuole induction during p21 suppression

**Fig. 2:**
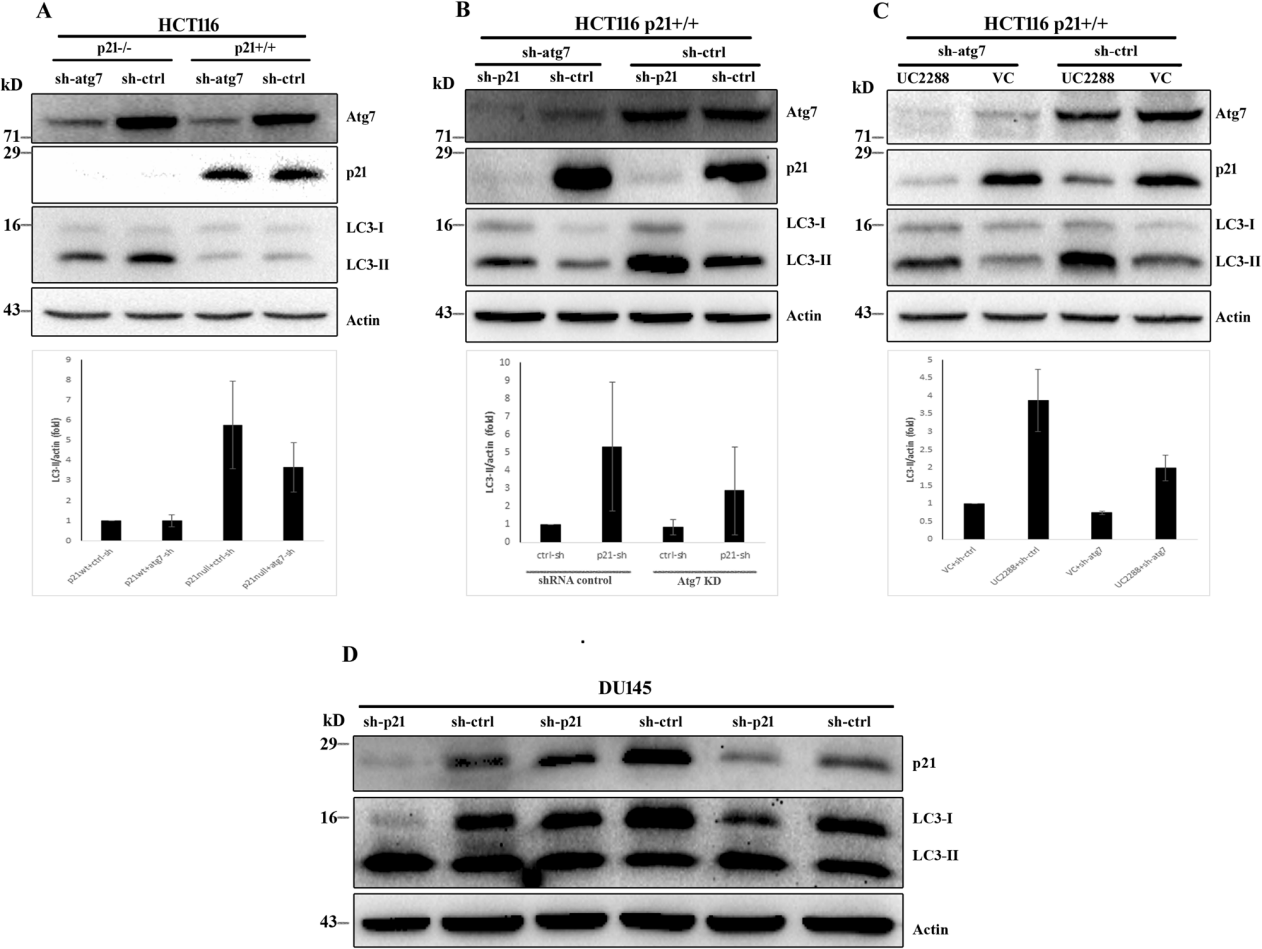
Atg7 knockdown prevents p21 suppression-dependent increased LC3 turnover in autophagy-competent cells. **A** Isogenic p21^+/+^ and p21^−/−^ HCT116 cells were stably transduced with scrambled or Atg7 targeting shRNA and analyzed by western blotting for LC3 conversion. **B**, **C** HCT116 p21wt cells, with stable expression of control and Atg7 shRNA, were subjected to p21 inhibition either by RNA silencing (**B**) or UC2288 (**C**) followed by immunoblot detection of LC3-I and II. **D** Western blot analysis of LC3-I/II in three biological replicates of DU145 cell lysates after transduction with control or p21 shRNA.

To determine the mechanism underlying enhanced autophagic vacuolization after p21 suppression, HCT116 p21wt and null cells were stably transduced with lentiviral particles encoding shRNA specific for an essential autophagy regulatory protein, Atg7. Knockdown efficacy of the candidate shRNA was examined by western blot assay showing substantial reduction of Atg7 expression in comparison to the control cells with stable expression of scrambled target sequence (Fig. 2A–C). Our results revealed that enhanced LC3-II level, in absence of p21, was suppressed by Atg7 depletion (Fig. 2A). Similar results were obtained when the HCT116 wild-type parental cells were transduced with p21-specific shRNA (Fig. 2B) or treated with UC2288 (Fig. 2C) and subjected to autophagy suppression by Atg7 silencing. To further validate the effect, we next used DU145 prostate cancer cells, which lacks functional autophagy due to the absence of Atg5 [23]. Suppression of p21 by gene silencing failed to activate LC3 lipidation in autophagy-compromised DU145 cells (Fig. 2D). These data suggested that induction of autophagic vacuoles after p21 inhibition follows the canonical pathway of autophagy.

**Fig. 3:**
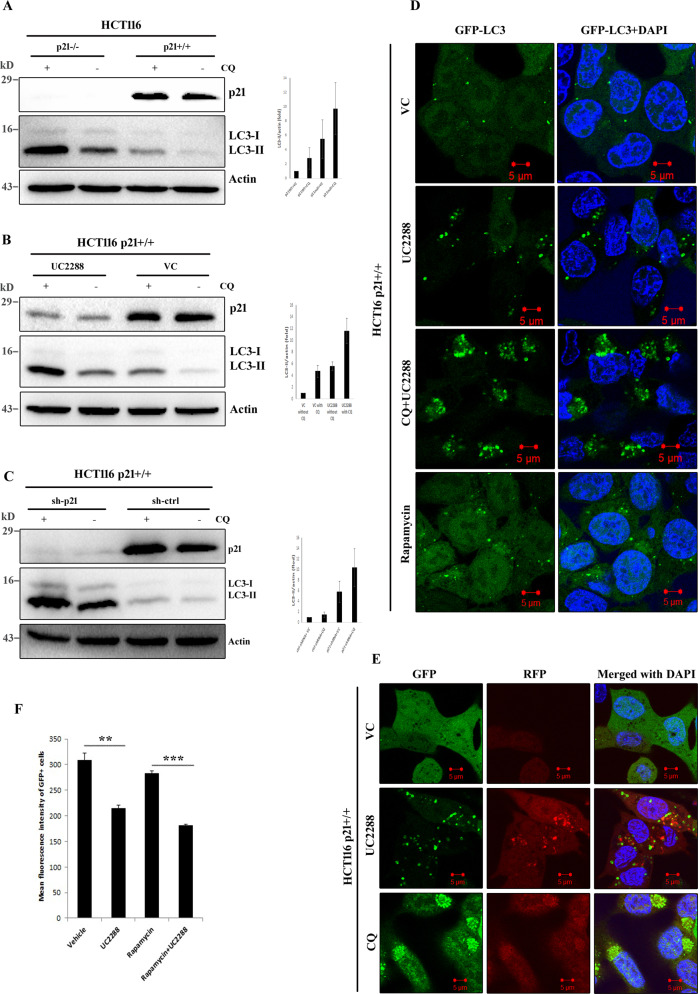
p21 inhibition induces autophagy flux. **A** HCT116 p21wt and null cells were incubated with 5 µM CQ for 24 h. Cell lysates were processed for western blot analysis with indicated antibodies. Relative LC3-II expression level among experimental groups were quantified from cell lysates of three independent experiments and graphically represented as mean ± SE. **B** Analysis of LC3 by immunoblotting in HCT116 p21^+/+^ cells treated with vehicle or UC2288 in the presence or absence of 5 µM CQ (2 h pre-incubation). Relative LC3-II expression level among experimental groups were quantified from cell lysates of three independent experiments and graphically represented as mean ± SE. **C** HCT116 p21^+/+^ cells were transiently transduced for 24 h with control or p21-specific shRNAs and incubated with or without 5 µM CQ for an additional 24 h before being analyzed for LC3 conversion by western blot assay. Relative LC3-II expression level among experimental groups was quantified from cell lysates of three independent experiments and graphically represented as mean ± SE. **D** Representative confocal micrographs of GFP-LC3 expressing HCT116 p21^+/+^ cells treated with vehicle or UC2288 in the presence or absence of 5 µM CQ (2 h pre-incubation). Rapamycin (2 µM for 24 h incubation) was used as the standard autophagy inducer in this study. **E** HCT116 p21^+/+^ cells, with stable expression of GFP-LC3-RFP-LC3ΔG, were treated with UC2288 after 2 h pre-incubation with 5 µM CQ and examined under confocal microscope. **F** GFP-LC3 expressing HCT116 p21wt cells were incubated with UC2288 alone or in combination with rapamycin. Cells were then analyzed by flow cytometry to measure GFP fluorescence intensity. Data presented as the mean of three (*n* = 3) independent experiments ± SE. **P < 0.01, ***P < 0.001.

Guided by above results, we investigated autophagic flux by different methods to distinguish higher autophagic sequestration, indicative of activation of conventional autophagy pathway, from decreased degradation of autophagic cargo in p21 inhibited cells. Toward this end, we first used lysosomal inhibitor chloroquine (CQ) and examined LC3-II turnover before and after p21 inhibition. Western blot experiment with whole cell lysates revealed additional induction of LC3-II in CQ-treated HCT116 p21null cells than vehicle-treated control (Fig. 3A). Likewise, pre-incubation of HCT116 p21wt cells with CQ caused marked enhancement of LC3-II upon neutralization of p21 activity either by UC2288 (Fig. 3B) or target specific shRNA (Fig. 3C). In agreement with the immunoblotting data, confocal microscopic analysis of GFP-LC3 expressing HCT116 p21wt cells showed much higher accumulation of GFP-LC3 puncta in CQ and UC2288 co-treatment group than the cells that were treated with UC2288 (Fig. 3D). These results suggested that enhancement of autophagy markers in p21 attenuated cells is indeed due to induction of autophagy rather than inhibition of autophagosome-lysosome fusion. To further validate p21 attenuation driven delivery of autophagosomal membranes into lysosomes, we used GFP-LC3-RFP-LC3ΔG fluorescence probe encoding both conjugation proficient and deficient LC3 that are tagged with GFP and RFP, respectively. The quenching of GFP fluorescence in autophagolysosomes and unchanged intensity of lipidation compromised RFP-LC3ΔG results in an increased RFP/GFP ratio during autophagic process [16]. Here, analysis of GFP-LC3-RFP-LC3ΔG expressing HCT116 p21wt cells by confocal microscopy revealed diffuse pattern of green and red fluorescence in vehicle-treated control cells. The GFP intensity was apparently decreased upon UC2288 treatment and was associated with appearance of distinct GFP and RFP puncta. The effect was reversed when the fusion of autophagosome with lysosome was blocked by incubating cells with CQ (Fig. 3E). Lysosomal degradation of LC3, indicative of fusion of autophagosome with lysosome, due to p21 depletion was also evident in flow cytometric analysis of GFP-LC3 expressing HCT116 cells. Here, incubation of cells with UC2288 or rapamycin (standard autophagy inducer) for 24 h resulted in substantial decrease in GFP fluorescence intensity in comparison to the untreated control group (Fig. 3F) and thereby indicated activation of functional autophagy. Altogether, these data confirm that p21 inhibits autophagy at physiological concentration.

### p21 modulates autophagy via p53 independent mechanisms

**Fig. 4:**
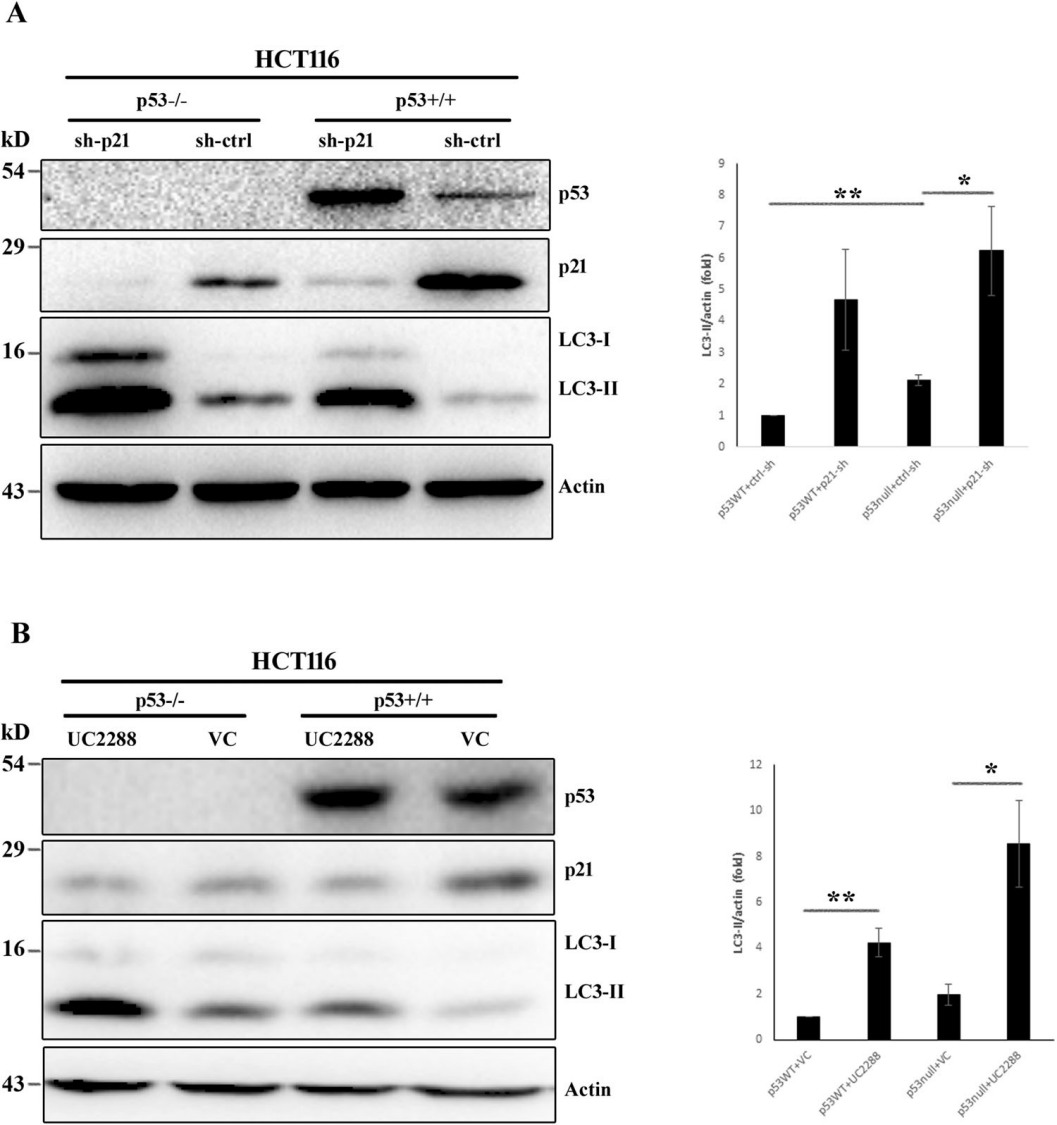
p53 independent autophagy modulation by p21. HCT116 p53wt and null cells were subjected to p21 attenuation either by sh-p21 lentivirus transduction (**A**) or by treating with UC2288 (**B**). Cells were then harvested and immunoblotting was carried out to determine relative expression of p53, p21, and LC3. Actin was probed as a normalization control. Densitometric analysis for LC3-II expression of three independent experiments was performed by ImageJ and represented graphically as mean ± SE. *P < 0.05. **P < 0.01.

In spite of being a bona fide downstream target, p21 has also been shown as a negative regulator of p53 [24] which in turn is known to modulate autophagy [25, 26]. Therefore, it is necessary to determine whether or not the autophagy modulatory effect of p21 is p53 dependent. To explore this, we used HCT116 p53 wild-type (p53^+/+^) and its isogenic null (p53^−/−^) cell lines in our study. The activity of p21 was suppressed, in these cell lines, either by treatment with UC2288 or by transducing with lentiviral particles encoding p21-specific shRNA. Absence of functional p53 in null cells was validated by western blot assay (Fig. 4A, B). Likewise, reduction in p21 level, upon pharmacological inhibition or genetic depletion, was confirmed by immunoblotting (Fig. 4A, B). In agreement with the previous report [26], enhancement of biochemical signs of autophagy (i.e., LC3-II level) was evident in p53 deficient cells as compared to the parental control cell line (Fig. 4A, B). Similarly, depletion of p21 resulted in increased LC3-II expression (Fig. 4A, B) which is in accordance with our earlier observation. However, a combination of p53 knockout and p21 attenuation, either by gene silencing (Fig. 4A) or by a pharmacological inhibitor (Fig. 4B), caused substantial induction of LC3-II turnover than the p53 deficient cells with a physiological level of p21 expression and thereby indicating that autophagy induction, upon p21 suppression, is mediated through p53 independent manner.

### Akt activation correlates with ROS-induced autophagy in p21 attenuated cells

**Fig. 5:**
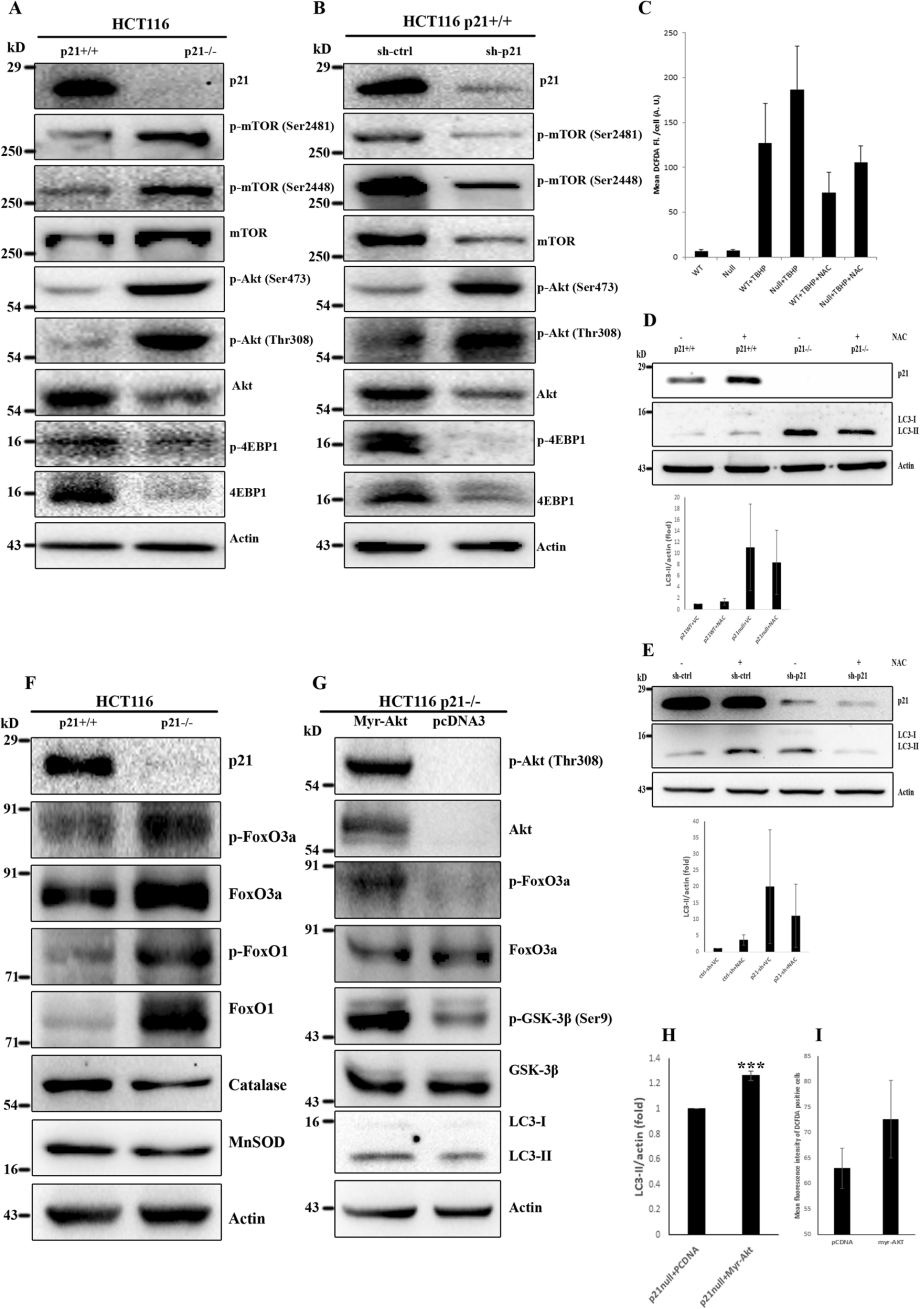
p21 inhibition induces autophagy by triggering intracellular ROS through activation of Akt kinase. **A** Lysates from HCT116 p21wt and null cells were resolved and immunoblotted with indicated Akt-mTOR signaling antibodies. **B** HCT116 p21^+/+^ cells were transiently transduced with lentiviral particles encoding control and p21-specific shRNA. Cell lysates were analyzed by western blotting using same sets antibodies as mentioned in panel **A**. **C** HCT116 p21wt and null cells were incubated with or without 250 µM tBHP for 24 h in the presence or absence of NAC (2.5 mM NAC; 4 h pre-incubation). Cells were then stained with 5 µM CM-H_2_DCFDA at 37 °C for 30 min in dark and analyzed by flow cytometry. Data presented as mean of three (*n* = 3) independent experiments ± SE. **D** HCT116 p21wt and null cells were incubated with or without 2.5 mM NAC for 4 h followed by immunoblot detection after probing with indicated antibodies. Relative LC3-II expression level among experimental groups was quantified from cell lysates of three independent experiments and graphically represented as mean ± SE. **E** HCT116 p21^+/+^ cells were subjected to p21 suppression by sh-p21 lentivirus transduction and co-incubated (for 4 h) with or without 2.5 mM NAC. The cells were then lysed and immunoblotting was carried out to determine relative conversion of LC3 as well as to confirm the silencing of p21 by gene-specific shRNA. Relative LC3-II expression level among experimental groups was quantified from cell lysates of three independent experiments and graphically represented as mean ± SE. **F** Representative immunoblots of HCT116 p21wt and null cell lysates showing increased phosphorylation of Akt substrates and reduced expression of ROS scavengers in p21 deficient cells. **G** HCT116 p21null cells were transiently transfected with plasmids encoding or not constitutively activated (myr) Akt for 48 h. Cell lysates were then immunoblotted with indicated antibodies to determine Akt activation and its effect on cellular autophagy. **H** Quantification of LC3-II turnover by ImageJ in pCDNA and Myr-Akt transfected cells of three independent experiments (mean ± SE; ***P < 0.001). **I** HCT116 p21null cells, with or without transient expression of myr-Akt, were analyzed by flow cytometry after 30 min staining with 5 µM CM-H_2_DCFDA.

To further increase mechanistic understanding on augmented autophagy in p21 deficient cells, we investigated Akt/mTOR pathway which is considered as major modulator of autophagy by sensing cellular energy status and negatively regulates it [27]. Contrary to the common notion, marked increase in Akt phosphorylation (Ser 473 and Thr 308), indicative of intrinsic kinase activity, was observed in p21 deficient cells (Fig. 5A). Correspondingly, p21 depletion was associated with enhanced phosphorylation of mTOR at Ser 2448 and Ser 2481 (Fig. 5A). Interestingly, in contrast to increased Akt and mTOR kinase activity, phosphorylation of 4E-BP1 (Thr 37/38), a downstream target of mTOR, was decreased in p21null cells (Fig. 5A). In agreement with the observations in p21 wild-type and null isogenic cells, silencing of p21 in HCT116 p21^+/+^ cells resulted in striking increase in Akt phosphorylation at Ser 473 and Thr 308 (Fig. 5B). However, RNAi mediated p21 knockdown led to reduced phosphorylation of mTOR and its direct substrate 4E-BP1 (Fig. 5B). The steady upregulation of phosphorylated Akt among inconsistent observations in p21 knocked down and knock out cells, led us to postulate that overactive Akt might be an essential player in triggering autophagy [28] in p21 attenuated cells which might act by inducing ROS [29, 30].

To explore this possibility, we compared intracellular ROS level between p21wt and null cells. Flow cytometric analysis of CM-H2DCFDA stained cells revealed marginally enhanced ROS in p21 deficient cells as compared to p21 proficient group at basal level (mean fl intensity 6.73 vs 6.17) as well as in response to tert-Butyl Hydroperoxide (tBHP) induced oxidative stress (mean fl intensity 186.14 vs 127.11) (Fig. 5C). Next, we investigated whether minimal increase in intracellular ROS has any role on enhanced autophagy level in p21 depleted cells. To this end, cells were exposed to ROS scavenger NAC and cell lysates were analyzed by immunoblotting for LC3-II turnover. As shown in Fig. 5D, treatment of cells with NAC resulted in marked decrease in LC3-II level in p21 deficient cells. The effect was further validated by silencing p21 in wild-type cells and determining LC3 turnover in the presence and absence of NAC. As can be seen in Fig. 5E, NAC pretreatment caused marked reduction in p21 suppression-dependent enhanced LC3-II level. Our data also validated ROS-dependent induction of autophagy in HCT116 cells by demonstrating tBHP driven additionally enhanced LC3-II level in both p21wt and null cells which was further decreased upon NAC treatment (Supplementary figure 2). Thus, it is likely that constitutively active Akt caused a persistent slight increase in intracellular ROS which was enough to trigger autophagy in p21 deficient cells.

In the following experiments, we sought to determine how Akt activity elevates intracellular ROS. Inhibitory phosphorylation of FoxO family transcription factors, downstream effectors of Akt, has been demonstrated to be a potential mechanism by which Akt directly regulates ROS level [31]. Here, we first examined the expression and post-translational modification of FoxO1 and FoxO3a in p21wt and null cells. Western blot analysis of cell lysates revealed an increase in total as well as phosphorylated FoxO3a (Ser 253) in p21^−/−^ cells (Fig. 5F). Similarly, we also observed upregulation of total and p-FoxO1 (Ser 256) in p21 deficient cells in comparison to the p21 proficient control groups (Fig. 5F). Nuclear exclusion of FoxO, due to Akt catalyzed phosphorylation, results in transcriptional repression of cellular anti-oxidant enzymes and eventual upregulation of ROS [29]. In accordance with the inhibitory phosphorylation of FoxO transcription factors, expression of their target ROS scavengers, catalase, and MnSoD, was suppressed in p21^−/−^ cells than isogenic p21^+/+^ cells (Fig. 5F). Thus, it appears that impaired radical scavenging might have led to augmentation of ROS in p21 deficient cells. To further determine putative involvement of Akt in the augmentation of intracellular ROS upon p21 deficiency, HCT116 p21^−/−^ cells were transiently transfected with empty vector or a constitutively active myr-Akt construct. Hyperactivation of Akt was confirmed by enhanced phosphorylation at Thr 308 residue as well as increased phosphorylation of downstream substrates such as GSK-3β and FoxO3a (Fig. 5G). Here, we found that hyperactivation of Akt triggered intracellular ROS in p21null cells (Fig. 5I) and thereby instigated significant induction of autophagy (Fig. 5H). Taken together, above results suggest that p21 suppression activates Akt kinase which in turn elevates intracellular ROS through inhibitory phosphorylation of FoxO transcription factors to trigger autophagy.

### Significance of autophagy on physiological role of p21 in cell proliferation

**Fig. 6:**
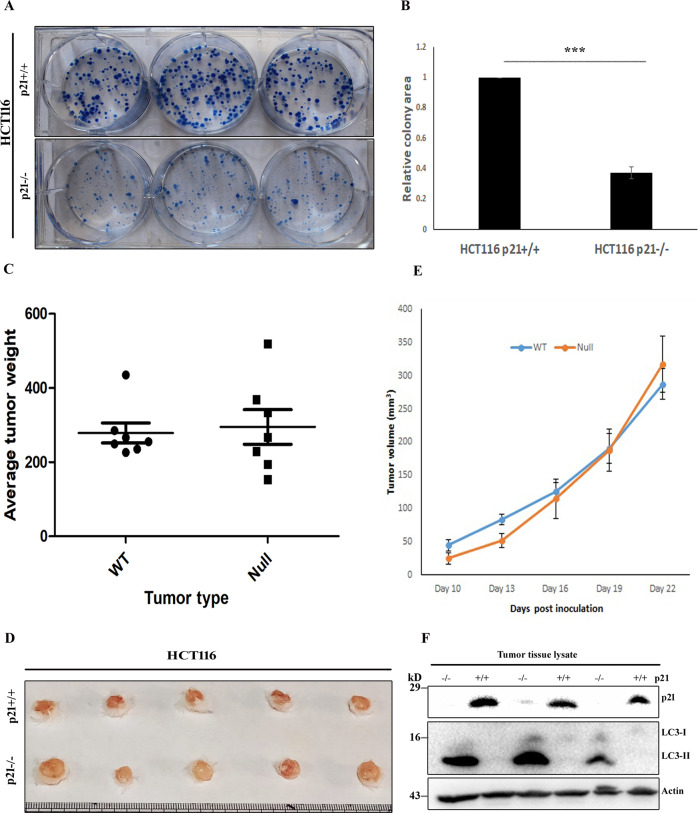
p21 modulated autophagy affects in vivo tumor growth kinetics. **A** Approximately 500 numbers of HCT116 p21^+/+^ and p21^−/−^ cells per well were grown in a six-well plate for 2 weeks, thereafter fixed and stained with crystal violet. **B** Quantitative analyses of colony area by ImageJ. (mean ± SE; ***P < 0.001) **C**, **D** NOD/SCID mice were subcutaneously injected individually with equal numbers (5 × 10^6^) of HCT116 p21wt and null cells at the right and left flank, respectively. Tumors were allowed to grow till 3 weeks after which the mice were sacrificed to dissect the tumors (**D**) for weighing (**C**); symbols represent individual mouse; data presented as mean ± SE. **E** Tumor size were recorded at regular intervals during the study and their volumes were calculated as described in Methods section. (*n* = 7 per group; mean ± SE are shown). **F** Tumor tissue lysates were analyzed by immunoblotting to confirm p21 expression in respective xenografts and determine relative conversion of LC3.

Although mutation of p21 is extremely rare in human malignancies, its influence has been recorded on wide variety of cancers including renal cancer, breast cancer, pancreatic cancer, testicular cancer, ovarian cancer, cervical cancer, squamous cell carcinomas and prostate cancer [32]. Therefore, it is pertinent to investigate the effect of p21 modulation on cancer cell proliferation and monitor differential (if any) progression of in vivo tumors derived from cancer cells with altered p21 level. In the present study, we first performed colony formation assay to examine the influence of p21 on growth and survival of eukaryotic cells. In contrast to the common perception of p21 as critical factor in halting cellular proliferation, results of our clonogenic assay revealed lesser number of viable colonies with reduced size in p21null cells in comparison to the p21wt cells (Fig. 6A, B). Similarly, incubation of HCT116 p21wt cells with UC2288 resulted in significant decrease in viable cell colonies as compared to the (vehicle) control groups (Supplementary figure 3A, B). To further validate predictive value of clonogenic assay on in vivo tumor growth potential, HCT116 xenografts were established in immunodeficient (NOD/SCID) mice by implanting p21wt and null cells into right and left flank, respectively and growth kinetics of corresponding tumors were monitored for 3 weeks until the animals become moribund. As shown in Fig. 6C, D, no obvious differences in average size and weight of harvested tumors between p21wt and null xenografts was observed at the end of the study. Similarly, mean volumes of both p21 proficient and deficient tumors were nearly equal, with marginally higher average volume of null xenografts, at the end of the study (Fig. 6E). All experimental animals appeared healthy at the time of euthanasia (Supplementary figure 3C). However, there were noticeable variances in growth kinetics during initial period progression. In agreement with in vitro clonogenic assay data, tumors with p21null background grew little slowly, than their wild-type counterparts, during earlier stage of development. But they (p21null tumors) progressed at much faster rate at approximately 2 weeks post inoculation and resembled those of wild-type xenografts in size and weight at the time of harvesting (Fig. 6E). Differential p21 expression in isogenic xenografts and higher level of basal autophagy in p21null tumors was confirmed by analyzing tumor tissue lysates by western blot assay (Fig. 6F). To further obtain a conclusive notion on differential growth kinetics of tumor xenografts with altered p21 genetic background, HCT116 p21^−/−^ (left) and parental p21^+/+^ (right) cells were implanted subcutaneously to both flanks of each nude mouse which allows better tumor monitoring because of their lack of fur coat and the tumors were allowed to grow for a more extended period. Moreover, sample size of the study was also increased to minimize the impact of variability, if any, of experimental data. Here, faster growth rate of p21 deficient xenografts, after an initial sluggishness for around 2 weeks, was more evident than wild-type counterparts. Likewise, size and weight of the harvested tumors were apparently higher in p21null xenografts than p21wt tumors (Supplementary figure 4). Altogether, above observations are in accordance with the theory that autophagy suppresses tumorigenesis during initial stages of development while it supports tumor cell growth and survival, by coping with intracellular and extracellular stresses, in established cancer [33, 34].

## Discussion

In tumor context, p21 has “antagonistic duality” towards cancer progression. Early studies have shown it to have anti-oncogenic potential owing to its ability to inhibit CDKs and inducing cellular senescence which is assumed to be mediated by nuclear-localized p21. Paradoxically, later studies have demonstrated it to exhibit oncogenic activities which are supported by findings that p21 is over-expressed in variety of human cancers including prostate, cervical, renal, breast, head and neck etc. [32]. The pro-oncogenic functions are believed to be mediated by cytoplasmic p21 through inhibitory binding with cytosolic pro-apoptotic proteins including caspases, Ask1, JNK etc. [12, 35] as well as by promoting assembly of cyclindD-CDK4/6 complex [36–38] and ability to repair DNA in tumor cells [39]. Moreover, there are emerging evidences pointing towards a fundamental role of p21 on modulation of autophagy albeit with contradictory functions [40–43]. In this context, it is quite likely that the ability of p21 to modulate autophagy may also contribute to its “antagonistic duality” in cancer progression. However, most of these studies projected p21 as an associated factor in modulation of autophagy by various pharmacological agents. Hence, direct evidence on influence of p21 on cellular autophagic process needs to be explored. Here, we demonstrate direct association of p21 in the regulation of autophagy and its underlying molecular mechanisms.

In the present study we provide evidence that p21 negatively regulates autophagy pathway. Multiple approaches were taken to establish an inverse relationship between autophagy and p21 protein level. Likewise, modulation of autophagy was demonstrated both biochemically and morphologically by several assays. The expression of p21 was inhibited in HCT116 colon cancer cell line by deletion, depletion, and pharmacological inhibition. In all instances, downregulation (or absence) of p21 resulted in substantial induction of autophagy. However, previous studies suggested an impairment of autophagy upon decrease in p21 expression [42, 43] in mouse heart tissues and cardio myocytes. Therefore, we sought to understand whether enhanced level of autophagy due to p21 inhibition is HCT116 cell line specific or this effect is a global phenomenon in cancer. To this end, p21 was depleted in a panel of cell lines of different human cancer origin. In agreement with the results observed in HCT116, downregulation of p21 was associated with induction of autophagy in all the other test cell lines. The outcome was further supported with the findings that restoration of p21 expression in null cells was associated with partial reduction of the upregulated LC3-II level.

We subsequently asked whether activation of autophagy due to p21 inhibition follow “classical” autophagy pathway. To address this, autophagy pathway was inhibited both at initiation stage as well as at later stage of autophagosome-lysosome fusion and monitored deviation, if any, from autophagy upregulation upon p21 depletion. Obstruction of autophagy initiation by depleting Atg7, one of the key elements in LC3 lipidation machinery, markedly neutralized augmentation of LC3-II turnover upon p21 inhibition. On the contrary, blockade of later events of autophagosome and lysosome fusion in canonical autophagy pathway caused more pronounced LC3-II turnover upon p21 attenuation suggesting enhanced autophagic sequestration. Alternatively, lysosomal degradation of autophagic cargo, in absence of any inhibitor, in p21 depleted cells was confirmed by using a probe containing both conjugation proficient and deficient LC3 that are tagged with two different fluorescent markers. This event was further quantitatively validated by carrying out GFP-LC3 degradation assay by flow cytometry. Most importantly, suppression of p21 failed to induce autophagy in DU145 cells which lacks functional Atg5 [23] and thereby autophagy incompetent. Altogether, these findings emphasize potential role of p21 in regulation of autophagy.

p21 serves as a major player in mediating tumor suppressive function of p53. Induction of p21 is considered to be a universal consequence in cells undergoing apoptosis in response to p53 activation. Moreover, there are emerging reports linking autophagy modulation to the tumor suppressive activity of p53. Restoration of p53 activity in p53 deficient cells has been shown to induce autophagy which may either promote cell death [44] or can protect cancer cells from apoptosis activators [45] in context-dependent manner. Conversely, attenuation of p53 has also been reported to activate autophagy [26]. Although the association of p21 and p53 in apoptosis signaling is well characterized, the influence of p53 on autophagy modulatory activity of p21 remain elusive. The possible association of p21 and p53 in the context of autophagy becomes more puzzling with our finding of upregulated p53 in p21 attenuated cells which is in agreement with previous reports demonstrating enhanced p53 stability upon p21 suppression [24, 46]. However, the observation that a combination of p53 knockout and p21 knockdown resulted in more pronounced conversion of LC3-I to LC3-II than any of these conditions, emphasizes p53 independent modulation of autophagy pathway by p21. It is quite likely that the apparent no/less impact of p53 on p21-dependent modulation of autophagy might be due to the loss of transcriptional activity of stabilized p53 to transactivate target genes in autophagy pathway in p21-depleted cells [46].

By virtue of its ability to critically coordinating numerous cellular metabolic, growth, and differentiation pathways, all of which are tightly linked with the cellular autophagic process, Akt/mTOR signaling cascade has emerged as master modulator of autophagy [47]. Akt-mTOR pathway negatively regulates autophagy and can be classically exemplified by amino acid deprivation mediated inhibition of Akt-mTOR signaling which in turn triggers autophagy to reutilize cellular constituents for fulfilling energy demand [48]. In agreement with this concept, our data showed downregulation of mTOR kinase activity, as evidenced with decreased phosphorylation at Ser 2448 and Ser 2481, upon p21 knockdown which might contribute in autophagy augmentation in p21 depleted cells. However, p21null cells showed higher post-translational modifications of mTOR than wild-type parental cells. Unexpectedly, we observed an increase in Akt phosphorylation (at Ser 473 and Thr 308), indicative of enhanced Akt kinase activity, both in p21 attenuated and deficient cells despite the fact that Akt negatively regulates autophagy [49]. This discrepancy may reflect potential mechanism of p21 deficient cells to overcome increased apoptosis sensitivity [50, 51] through activation of Akt [52]. However, sustained Akt hyperactivation in turn led to increase in intracellular ROS in p21-depleted cells. Our results demonstrated inhibitory phosphorylation of FoxO transcription factors and consequent downregulation of ROS scavengers [53, 54] as potential mechanism of Akt-dependent escalation of ROS [29]. Finally, enhanced ROS production, in p21 attenuated cells, led to induction of autophagy [55]. It is also reasonable to believe that accumulation of ROS due to p21 suppression may contribute to maintaining enhanced Akt activity [29].

The influence of p21 on tumorigenesis is not clear yet and is associated with variable outcomes. Several mechanisms have been proposed to explain contradictory roles of p21 in oncogenesis. While tumor suppressive role of p21, a bona fide p53 target gene, is primarily attributed to its canonical function in inducing cellular senescence and cell cycle arrest as “universal inhibitor” of cyclin kinases, many recent studies demonstrated oncogenic potential of cytosolic p21 through inhibition of apoptosis and activation of cyclin D–CDK4/6 complexes [39, 56–58]. To complicate things further, no straightforward clinical correlation exists between expression of p21 and human malignancies which eventually turned out to be context-dependent contradictory association of p21 in cancer [59]. Here, our finding that p21 depletion is associated with decreased in vitro cell proliferation supports the theory of pro-oncogenic activity of p21 at physiological concentration. The effect was further validated with our in vivo observation on higher growth kinetics of p21 wild-type tumor during initial 2 wks post transplantation. Interestingly, after around 14 days of tumor establishment, p21null xenografts grew at faster rate than their wild-type counterparts and thus uncovering an unexpected pattern of oncogenic impact of p21. It is quite likely that p21, as a physiological inhibitor of autophagy, positively modulates early events during tumor development [60]. However, augmented autophagy might have favored proliferation of p21 deficient cells at later stage in hypoxic and nutrient-deprived microenvironment in established tumors [61]. The results of our study are also in agreement with the notion that enhanced autophagy, upon p21 attenuation, suppresses cancer development during early stages but fuels their growth once a tumor is established [62].

In summary, the findings of our study provide a novel insight into the association between p21 and autophagy signaling which could impact tumor progression. Constitutive activation of Akt and ensuing ROS upregulation could be the major underlying mechanism in triggering autophagy upon p21 suppression.

## Supplementary information

Supplementary figure legends

Supplementary figure 1

Supplementary figure 2

Supplementary figure 3

Supplementary figure 4

Figure 1 Original Western blots

Figure 1G Remaining original Western blots

Figure 2A, B and C Original Western blots

Figure 2D Original Western blots

Figure 3 Original Western blots

Figure 4 Original Western blots

Figure 5A Original Western blots

Figure 5B Original Western blots

Figure 5D and E Original Western blots

Figure 5F Original Western blots

Figure 5G Original Western blots

Figure 6F Original Western blots

Checklist

## Data Availability

All experimental datasets generated and/or analyzed during the study are mostly included in this published article. Additional data are available from the corresponding author upon reasonable request.

## References

[1] Waga S, Hannon GJ, Beach D, Stillman B (1994). The p21 inhibitor of cyclin-dependent kinases controls DNA replication by interaction with PCNA. Nature.

[2] Xiong Y, Hannon GJ, Zhang H, Casso D, Kobayashi R, Beach D (1993). p21 is a universal inhibitor of cyclin kinases. Nature.

[3] el-Deiry WS (1998). p21/p53, cellular growth control and genomic integrity. Curr Top Microbiol Immunol.

[4] Fang L, Igarashi M, Leung J, Sugrue MM, Lee SW, Aaronson SA (1999). p21Waf1/Cip1/Sdi1 induces permanent growth arrest with markers of replicative senescence in human tumor cells lacking functional p53. Oncogene.

[5] Romanov VS, Rudolph KL (2016). p21 shapes cancer evolution. Nat Cell Biol.

[6] Wedam S, Fashoyin-Aje L, Bloomquist E, Tang S, Sridhara R, Goldberg KB (2020). FDA approval summary: palbociclib for male patients with metastatic breast cancer. Clin Cancer Res.

[7] Xie W, Zhou J. Aberrant regulation of autophagy in mammalian diseases. Biol Lett. 2018;14. 10.1098/rsbl.2017.0540 PMC5803588 29321247

[8] Liang XH, Jackson S, Seaman M, Brown K, Kempkes B, Hibshoosh H (1999). Induction of autophagy and inhibition of tumorigenesis by beclin 1. Nature.

[9] Azad MB, Chen Y, Gibson SB (2009). Regulation of autophagy by reactive oxygen species (ROS): implications for cancer progression and treatment. Antioxid Redox Signal.

[10] Degenhardt K, Mathew R, Beaudoin B, Bray K, Anderson D, Chen G (2006). Autophagy promotes tumor cell survival and restricts necrosis, inflammation, and tumorigenesis. Cancer Cell.

[11] Young AR, Narita M, Ferreira M, Kirschner K, Sadaie M, Darot JF (2009). Autophagy mediates the mitotic senescence transition. Genes Dev.

[12] Asada M, Yamada T, Ichijo H, Delia D, Miyazono K, Fukumuro K (1999). Apoptosis inhibitory activity of cytoplasmic p21(Cip1/WAF1) in monocytic differentiation. EMBO J.

[13] Chang BD, Watanabe K, Broude EV, Fang J, Poole JC, Kalinichenko TV (2000). Effects of p21Waf1/Cip1/Sdi1 on cellular gene expression: implications for carcinogenesis, senescence, and age-related diseases. Proc Natl Acad Sci USA.

[14] Zhao H, Jin S, Antinore MJ, Lung FD, Fan F, Blanck P (2000). The central region of Gadd45 is required for its interaction with p21/WAF1. Exp Cell Res.

[15] Fung C, Lock R, Gao S, Salas E, Debnath J (2008). Induction of autophagy during extracellular matrix detachment promotes cell survival. Mol Biol Cell.

[16] Kaizuka T, Morishita H, Hama Y, Tsukamoto S, Matsui T, Toyota Y (2016). An autophagic flux probe that releases an internal control. Mol Cell.

[17] Ramaswamy S, Nakamura N, Vazquez F, Batt DB, Perera S, Roberts TM (1999). Regulation of G1 progression by the PTEN tumor suppressor protein is linked to inhibition of the phosphatidylinositol 3-kinase/Akt pathway. Proc Natl Acad Sci USA.

[18] Hasanain M, Sahai R, Pandey P, Maheshwari M, Choyal K, Gandhi D (2020). Microtubule disrupting agent-mediated inhibition of cancer cell growth is associated with blockade of autophagic flux and simultaneous induction of apoptosis. Cell Prolif.

[19] Chin YR, Yuan X, Balk SP, Toker A (2014). PTEN-deficient tumors depend on AKT2 for maintenance and survival. Cancer Discov.

[20] Bhattacharjee A, Hasanain M, Kathuria M, Singh A, Datta D, Sarkar J (2018). Ormeloxifene-induced unfolded protein response contributes to autophagy-associated apoptosis via disruption of Akt/mTOR and activation of JNK. Sci Rep.

[21] Pandey P, Singh D, Hasanain M, Ashraf R, Maheshwari M, Choyal K (2019). 7-hydroxyfrullanolide, isolated from Sphaeranthus indicus, inhibits colorectal cancer cell growth by p53-dependent and -independent mechanism. Carcinogenesis.

[22] Wettersten HI, Hee Hwang S, Li C, Shiu EY, Wecksler AT, Hammock BD (2013). A novel p21 attenuator which is structurally related to sorafenib. Cancer Biol Ther.

[23] Hasanain M, Bhattacharjee A, Pandey P, Ashraf R, Singh N, Sharma S (2015). alpha-Solanine induces ROS-mediated autophagy through activation of endoplasmic reticulum stress and inhibition of Akt/mTOR pathway. Cell Death Dis.

[24] Broude EV, Demidenko ZN, Vivo C, Swift ME, Davis BM, Blagosklonny MV (2007). p21 (CDKN1A) is a negative regulator of p53 stability. Cell Cycle.

[25] Tasdemir E, Chiara Maiuri M, Morselli E, Criollo A, D’Amelio M, Djavaheri-Mergny M (2008). A dual role of p53 in the control of autophagy. Autophagy.

[26] Tasdemir E, Maiuri MC, Galluzzi L, Vitale I, Djavaheri-Mergny M, D’Amelio M (2008). Regulation of autophagy by cytoplasmic p53. Nat Cell Biol.

[27] Heras-Sandoval D, Perez-Rojas JM, Hernandez-Damian J, Pedraza-Chaverri J (2014). The role of PI3K/AKT/mTOR pathway in the modulation of autophagy and the clearance of protein aggregates in neurodegeneration. Cell Signal.

[28] Karim MR, Fisher CR, Kapphahn RJ, Polanco JR, Ferrington DA (2020). Investigating AKT activation and autophagy in immunoproteasome-deficient retinal cells. PLoS One.

[29] Nogueira V, Park Y, Chen CC, Xu PZ, Chen ML, Tonic I (2008). Akt determines replicative senescence and oxidative or oncogenic premature senescence and sensitizes cells to oxidative apoptosis. Cancer Cell.

[30] Nogueira V, Patra KC, Hay N (2018). Selective eradication of cancer displaying hyperactive Akt by exploiting the metabolic consequences of Akt activation. Elife.

[31] Dolado I, Nebreda AR (2008). AKT and oxidative stress team up to kill cancer cells. Cancer Cell.

[32] Liu R, Wettersten HI, Park SH, Weiss RH (2013). Small-molecule inhibitors of p21 as novel therapeutics for chemotherapy-resistant kidney cancer. Future Med Chem.

[33] Amaravadi RK, Kimmelman AC, Debnath J (2019). Targeting autophagy in cancer: recent advances and future directions. Cancer Discov.

[34] Mulcahy Levy JM, Thorburn A (2020). Autophagy in cancer: moving from understanding mechanism to improving therapy responses in patients. Cell Death Differ.

[35] Zhou BP, Liao Y, Xia W, Spohn B, Lee MH, Hung MC (2001). Cytoplasmic localization of p21Cip1/WAF1 by Akt-induced phosphorylation in HER-2/neu-overexpressing cells. Nat Cell Biol.

[36] Alt JR, Gladden AB, Diehl JA (2002). p21(Cip1) promotes cyclin D1 nuclear accumulation via direct inhibition of nuclear export. J Biol Chem.

[37] Cheng M, Olivier P, Diehl JA, Fero M, Roussel MF, Roberts JM (1999). The p21(Cip1) and p27(Kip1) CDK ‘inhibitors’ are essential activators of cyclin D-dependent kinases in murine fibroblasts. EMBO J.

[38] LaBaer J, Garrett MD, Stevenson LF, Slingerland JM, Sandhu C, Chou HS (1997). New functional activities for the p21 family of CDK inhibitors. Genes Dev.

[39] Abbas T, Dutta A (2009). p21 in cancer: intricate networks and multiple activities. Nat Rev Cancer.

[40] Capparelli C, Chiavarina B, Whitaker-Menezes D, Pestell TG, Pestell RG, Hulit J (2012). CDK inhibitors (p16/p19/p21) induce senescence and autophagy in cancer-associated fibroblasts, “fueling” tumor growth via paracrine interactions, without an increase in neo-angiogenesis. Cell Cycle.

[41] Fujiwara K, Daido S, Yamamoto A, Kobayashi R, Yokoyama T, Aoki H (2008). Pivotal role of the cyclin-dependent kinase inhibitor p21WAF1/CIP1 in apoptosis and autophagy. J Biol Chem.

[42] Huang S, Xu M, Liu L, Yang J, Wang H, Wan C (2020). Autophagy is involved in the protective effect of p21 on LPS-induced cardiac dysfunction. Cell Death Dis.

[43] Xu M, Wan CX, Huang SH, Wang HB, Fan D, Wu HM (2019). Oridonin protects against cardiac hypertrophy by promoting P21-related autophagy. Cell Death Dis.

[44] Crighton D, Wilkinson S, O’Prey J, Syed N, Smith P, Harrison PR (2006). DRAM, a p53-induced modulator of autophagy, is critical for apoptosis. Cell.

[45] Amaravadi RK, Yu D, Lum JJ, Bui T, Christophorou MA, Evan GI (2007). Autophagy inhibition enhances therapy-induced apoptosis in a Myc-induced model of lymphoma. J Clin Invest.

[46] Pang LY, Scott M, Hayward RL, Mohammed H, Whitelaw CB, Smith GC (2011). p21(WAF1) is component of a positive feedback loop that maintains the p53 transcriptional program. Cell Cycle.

[47] Saxton RA, Sabatini DM (2017). mTOR signaling in growth, metabolism, and disease. Cell.

[48] Kim YC, Guan KL (2015). mTOR: a pharmacologic target for autophagy regulation. J Clin Invest.

[49] Janku F, McConkey DJ, Hong DS, Kurzrock R (2011). Autophagy as a target for anticancer therapy. Nat Rev Clin Oncol.

[50] Lazzarini R, Moretti S, Orecchia S, Betta PG, Procopio A, Catalano A (2008). Enhanced antitumor therapy by inhibition of p21waf1 in human malignant mesothelioma. Clin Cancer Res.

[51] Wang YA, Elson A, Leder P (1997). Loss of p21 increases sensitivity to ionizing radiation and delays the onset of lymphoma in atm-deficient mice. Proc Natl Acad Sci USA.

[52] Franke TF, Hornik CP, Segev L, Shostak GA, Sugimoto C (2003). PI3K/Akt and apoptosis: size matters. Oncogene.

[53] Kops GJ, Dansen TB, Polderman PE, Saarloos I, Wirtz KW, Coffer PJ (2002). Forkhead transcription factor FOXO3a protects quiescent cells from oxidative stress. Nature.

[54] Nemoto S, Finkel T (2002). Redox regulation of forkhead proteins through a p66shc-dependent signaling pathway. Science.

[55] Filomeni G, De Zio D, Cecconi F (2015). Oxidative stress and autophagy: the clash between damage and metabolic needs. Cell Death Differ.

[56] Marhenke S, Buitrago-Molina LE, Endig J, Orlik J, Schweitzer N, Klett S (2014). p21 promotes sustained liver regeneration and hepatocarcinogenesis in chronic cholestatic liver injury. Gut.

[57] Romanov VS, Pospelov VA, Pospelova TV (2012). Cyclin-dependent kinase inhibitor p21(Waf1): contemporary view on its role in senescence and oncogenesis. Biochemistry.

[58] Weiss RH (2003). p21Waf1/Cip1 as a therapeutic target in breast and other cancers. Cancer Cell.

[59] Roninson IB (2002). Oncogenic functions of tumour suppressor p21(Waf1/Cip1/Sdi1): association with cell senescence and tumour-promoting activities of stromal fibroblasts. Cancer Lett.

[60] Biankin AV, Kench JG, Morey AL, Lee CS, Biankin SA, Head DR (2001). Overexpression of p21(WAF1/CIP1) is an early event in the development of pancreatic intraepithelial neoplasia. Cancer Res.

[61] Yang X, Yu DD, Yan F, Jing YY, Han ZP, Sun K (2015). The role of autophagy induced by tumor microenvironment in different cells and stages of cancer. Cell Biosci.

[62] Maes H, Rubio N, Garg AD, Agostinis P (2013). Autophagy: shaping the tumor microenvironment and therapeutic response. Trends Mol Med.

