# Supplementary material for: Inhibition of p21 activates Akt kinase to trigger ROS-induced autophagy and impacts on tumor growth rate
Source: Cell Death Dis. 2022 Dec 15;13(12):1045. doi: 10.1038/s41419-022-05486-1 (PMC9755229; doi:10.1038/s41419-022-05486-1)
Supplement: Supplementary file 1 — Supplementary figure legends [file 41419_2022_5486_MOESM1_ESM.docx]

**Supplementary figure legends:**

Supplementary figure 1. **Modulation of autophagy by p21.** (A) Confocal micrographs of vehicle and UC2288 treated HCT116 p21^+/+^ cells after immunostaining with anti-LC3 antibody. (B) Quantification of LC3 specific puncta (>25 cells) by ImageJ (***P*<0.01). (C) Complementation of the p21 gene neutralized augmented autophagy in HCT116 p21^-/-^ cells. LC3 and p21 levels were analyzed by immune blotting after conditional expression of dsRED tagged p21 gene in presence of doxycycline.

Supplementary figure 2. **ROS dependent induction of autophagy in HCT116 cells.** HCT116 p21wt and null cells were incubated for 24 h in presence or absence of 250 µM tBHP after 4 h pre-incubation with or without 2.5 mM NAC. Cell lysates were analyzed by western blotting after probing with indicated antibodies.

Supplementary figure 3. **Impact of p21 modulation on HCT116 cell growth *in vitro* and *in vivo*** (A) Clonogenic assay performed in HCT116 p21^+/+^ cells after incubation with or without UC228. (B) Total area of viable colonies were quantified by ImageJ, normalized with vehicle control group and represented graphically as mean± SE. ****P*<0.001 (C) *In vivo* growth of HCT116 xenografts with differential expression of p21 in NOD/SCID mice. HCT116 p21wt and null cells were inoculated subcutaneously at right and left flank, respectively of NOD/SCID mice. After 3 weeks post inoculation, the animals were euthanized and photographed.

Supplementary figure 4. **Comparative growth kinetics of HCT116 xenografts with different p21 genetic background in nude mice**. (A) Nude mice were engrafted with HCT116 p21wt and null cells at the right and left flank, respectively and tumor size were measured on every 3^rd^ day from 7 days post implantation. (B) Calculated tumor volumes during the course of the study. Data represented graphically as mean± SE (n=9 per group); **P*<0.05. (C) The animals were sacrificed at the end of the study on 25^th^ day post implation and (D) the tumors were harvested and (E) weighed (**P*<0.05). (F) Expression level of p21 and LC3-II turnover in tumor tissues was analysed by western blotting.
