# Supplementary figures and images for: Inhibition of p21 activates Akt kinase to trigger ROS-induced autophagy and impacts on tumor growth rate
Source: Cell Death Dis. 2022 Dec 15;13(12):1045. doi: 10.1038/s41419-022-05486-1 (PMC9755229; doi:10.1038/s41419-022-05486-1)

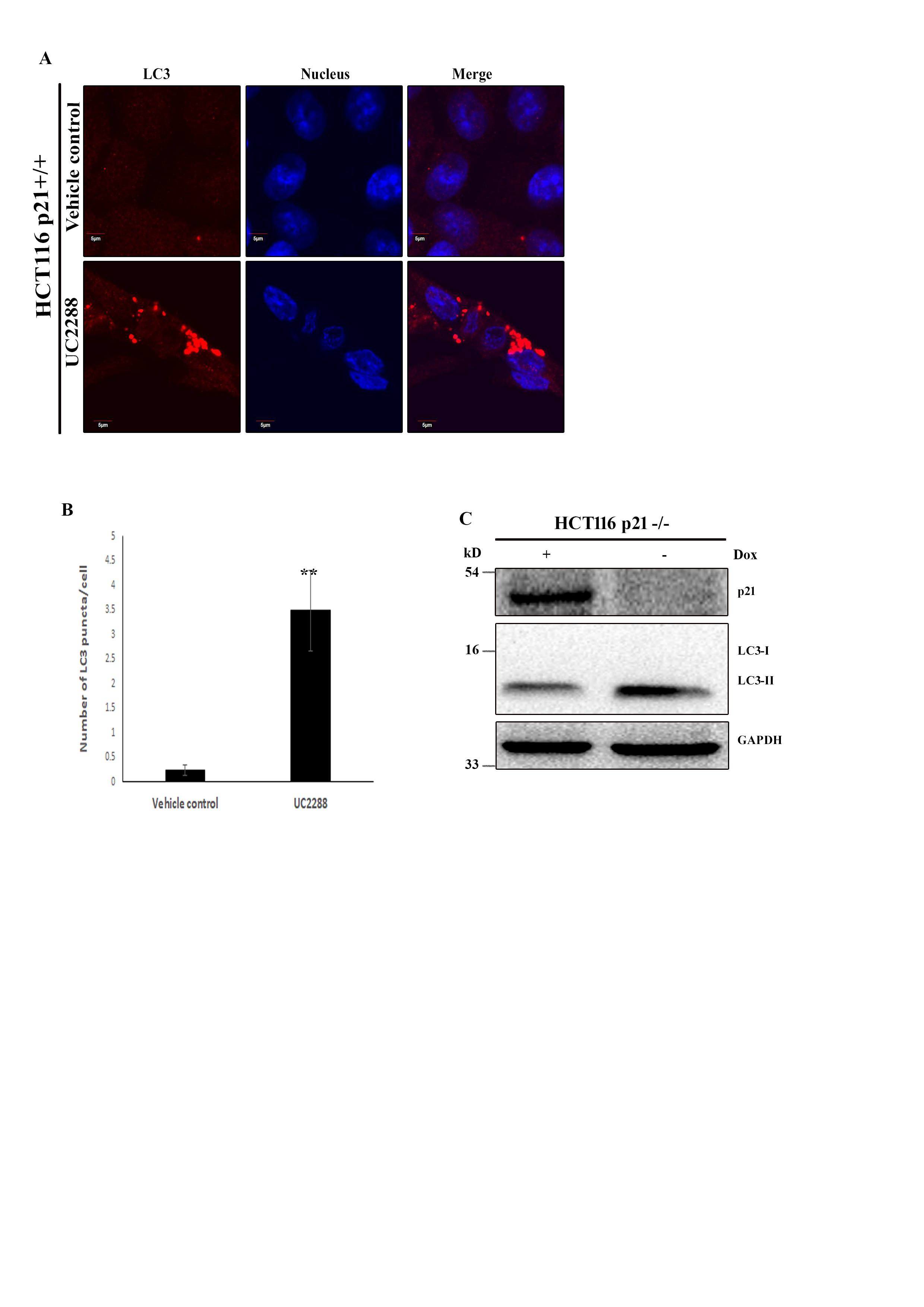

Supplement: Supplementary file 2 — Supplementary figure 1 [file 41419_2022_5486_MOESM2_ESM.tif]

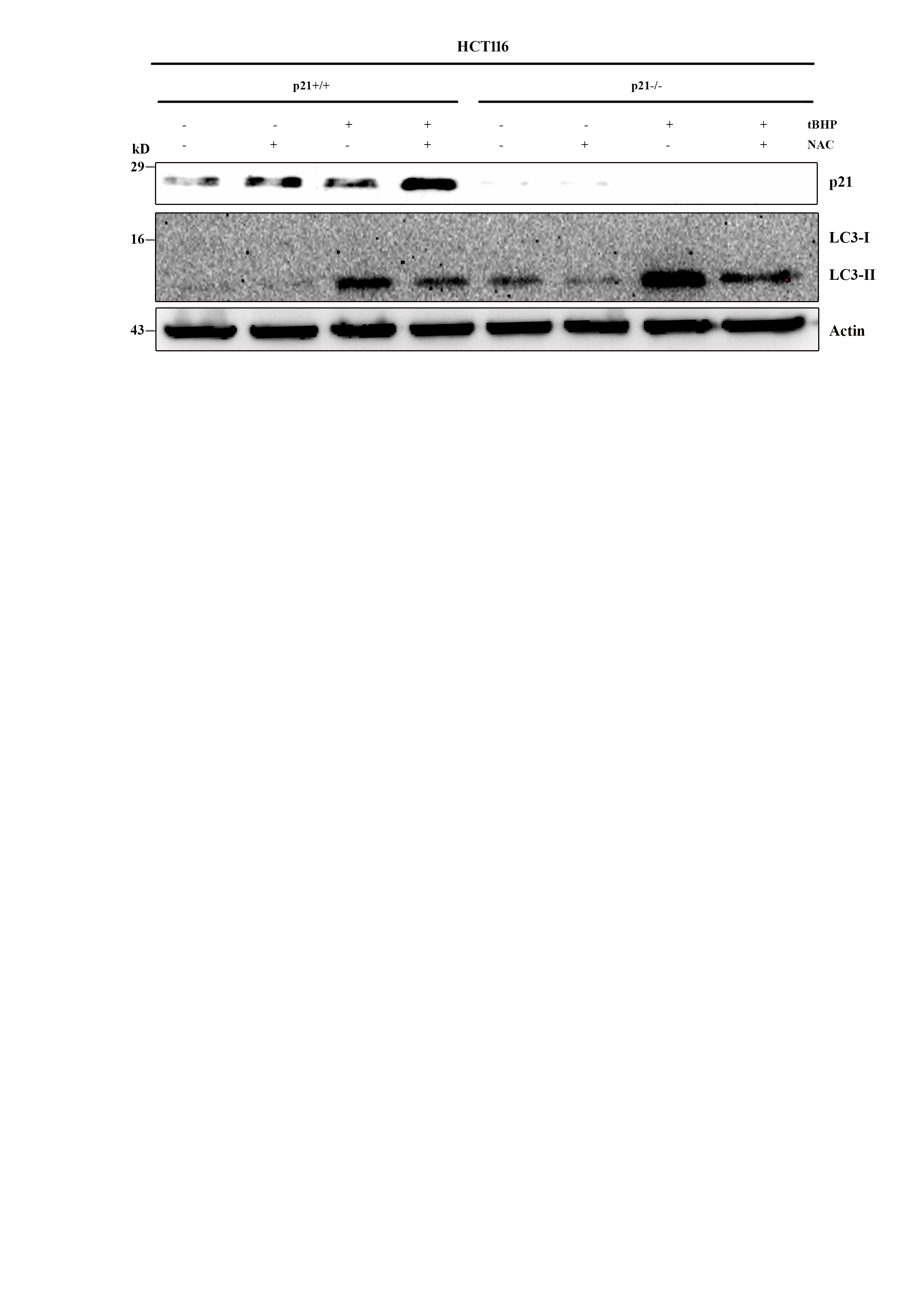

Supplement: Supplementary file 3 — Supplementary figure 2 [file 41419_2022_5486_MOESM3_ESM.tif]

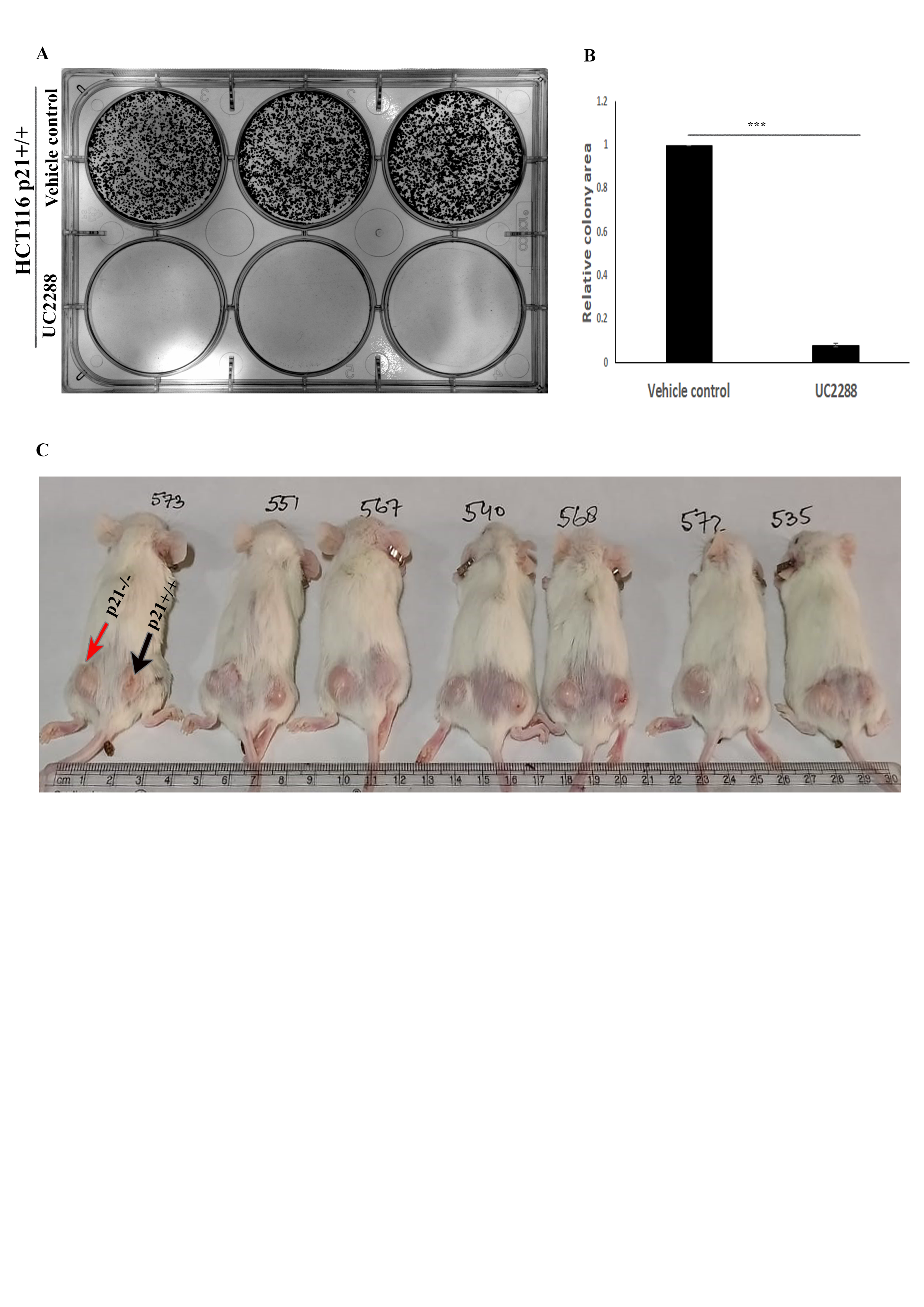

Supplement: Supplementary file 4 — Supplementary figure 3 [file 41419_2022_5486_MOESM4_ESM.tif]

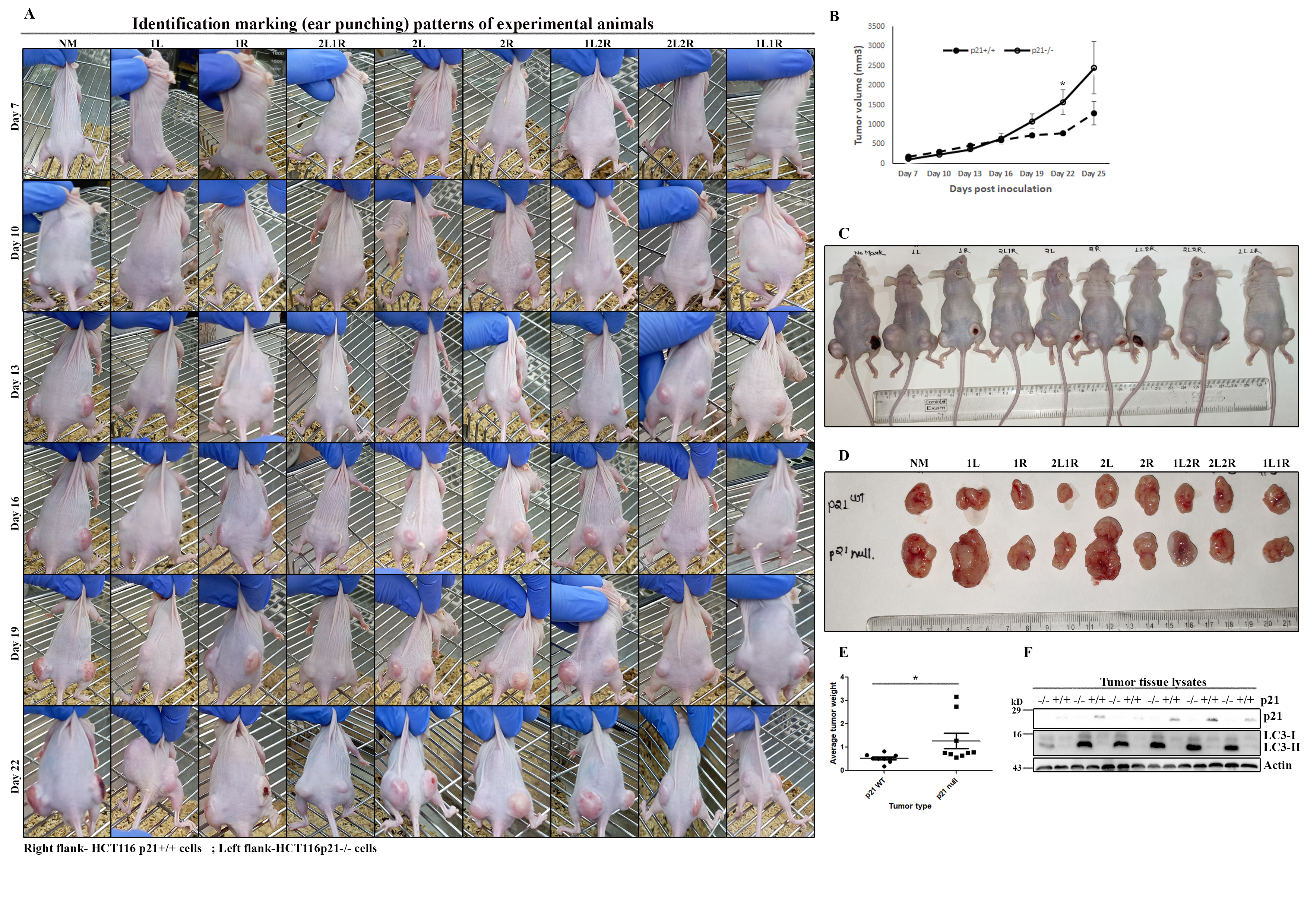

Supplement: Supplementary file 5 — Supplementary figure 4 [file 41419_2022_5486_MOESM5_ESM.tif]

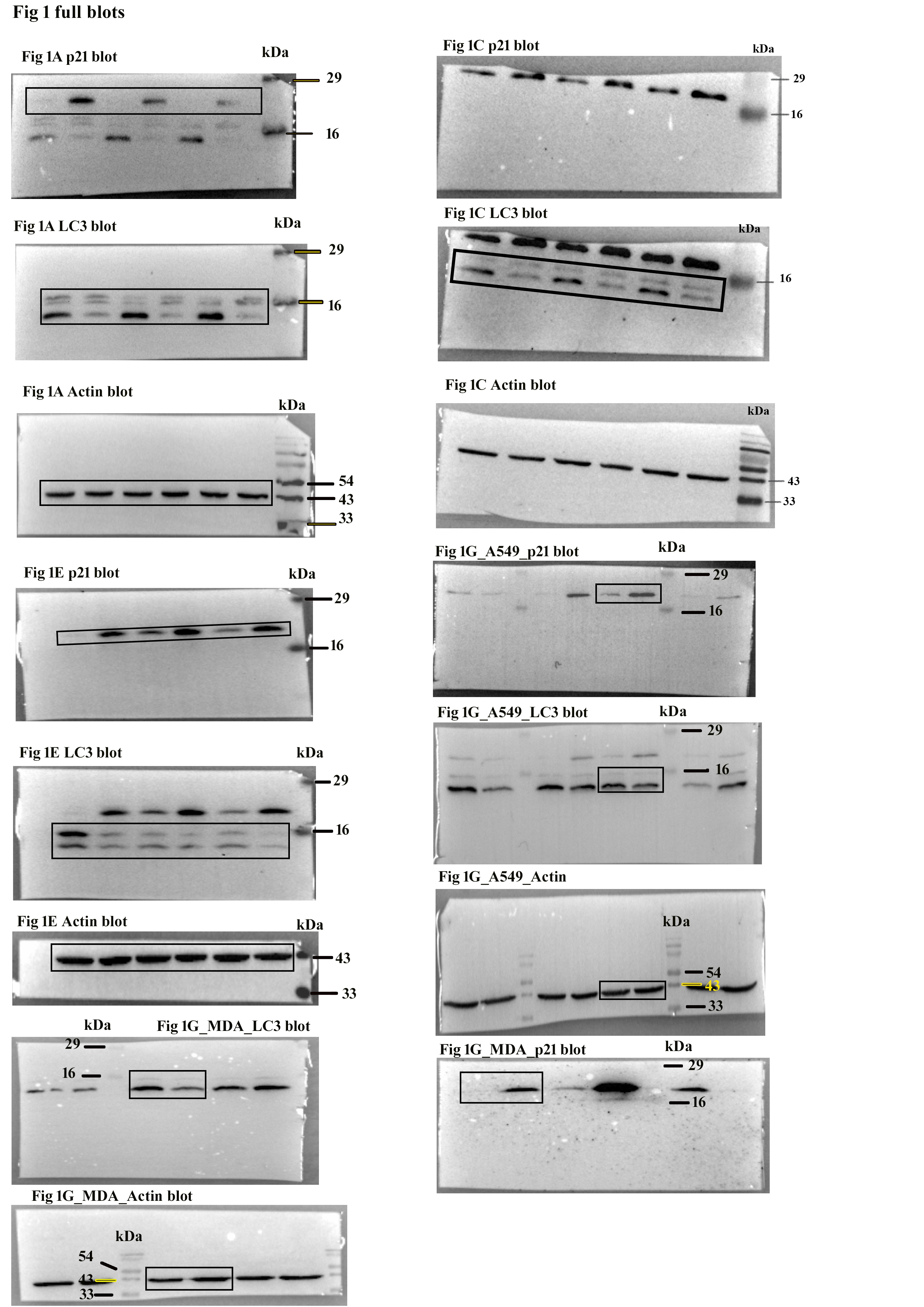

Supplement: Supplementary file 6 — Figure 1 Original Western blots [file 41419_2022_5486_MOESM6_ESM.tif]

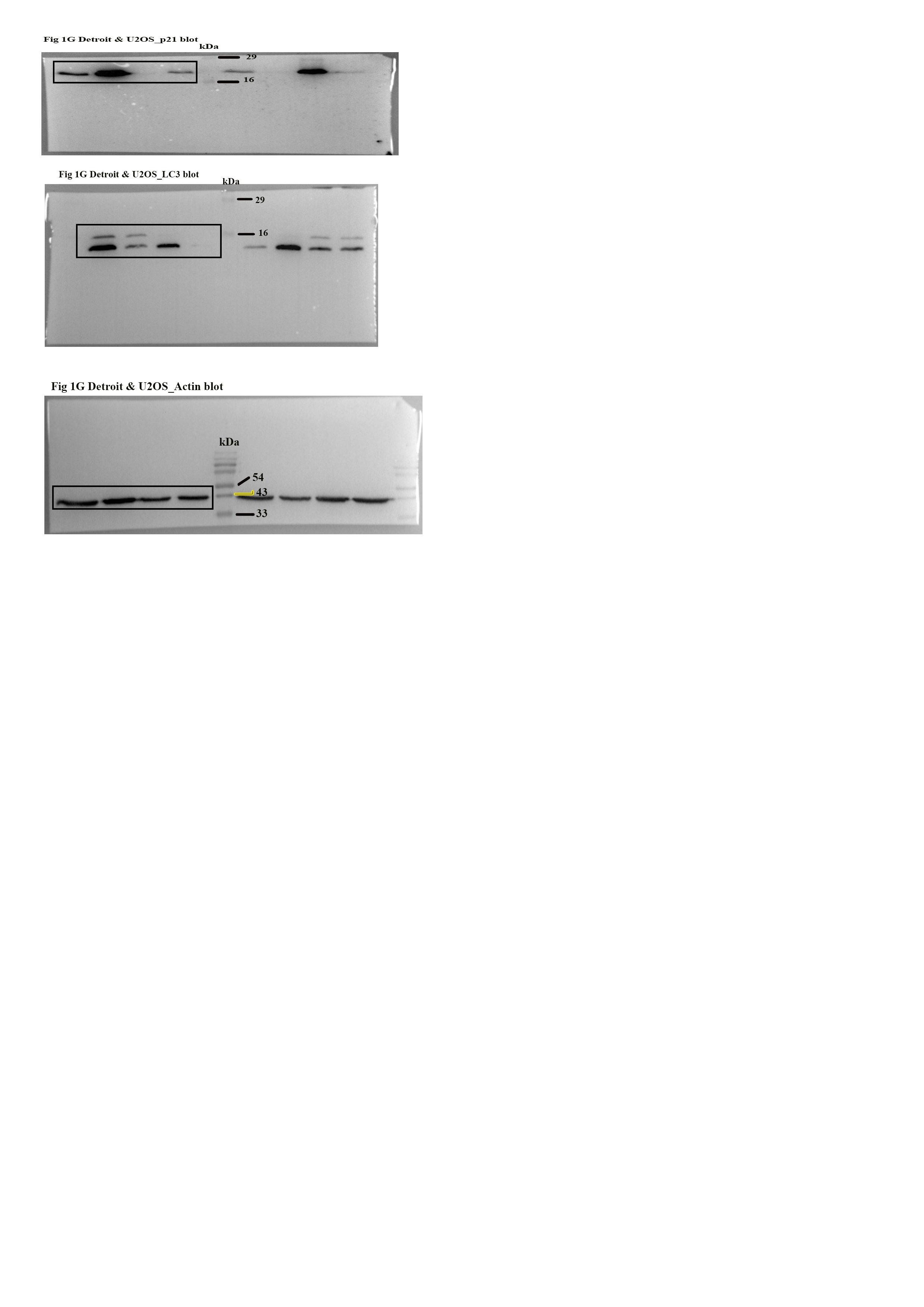

Supplement: Supplementary file 7 — Figure 1G Remaining original Western blots [file 41419_2022_5486_MOESM7_ESM.tif]

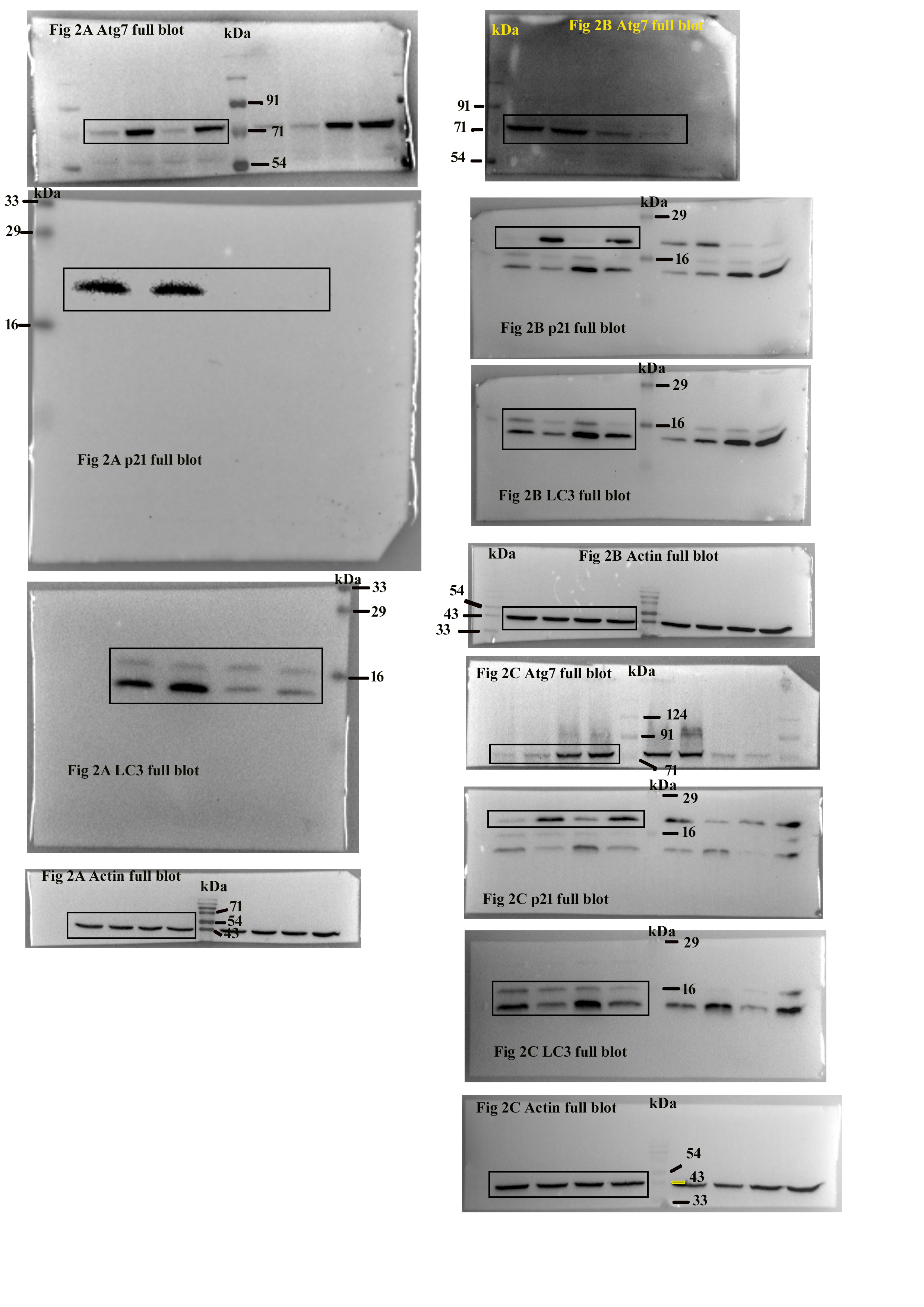

Supplement: Supplementary file 8 — Figure 2A, B and C Original Western blots [file 41419_2022_5486_MOESM8_ESM.tif]

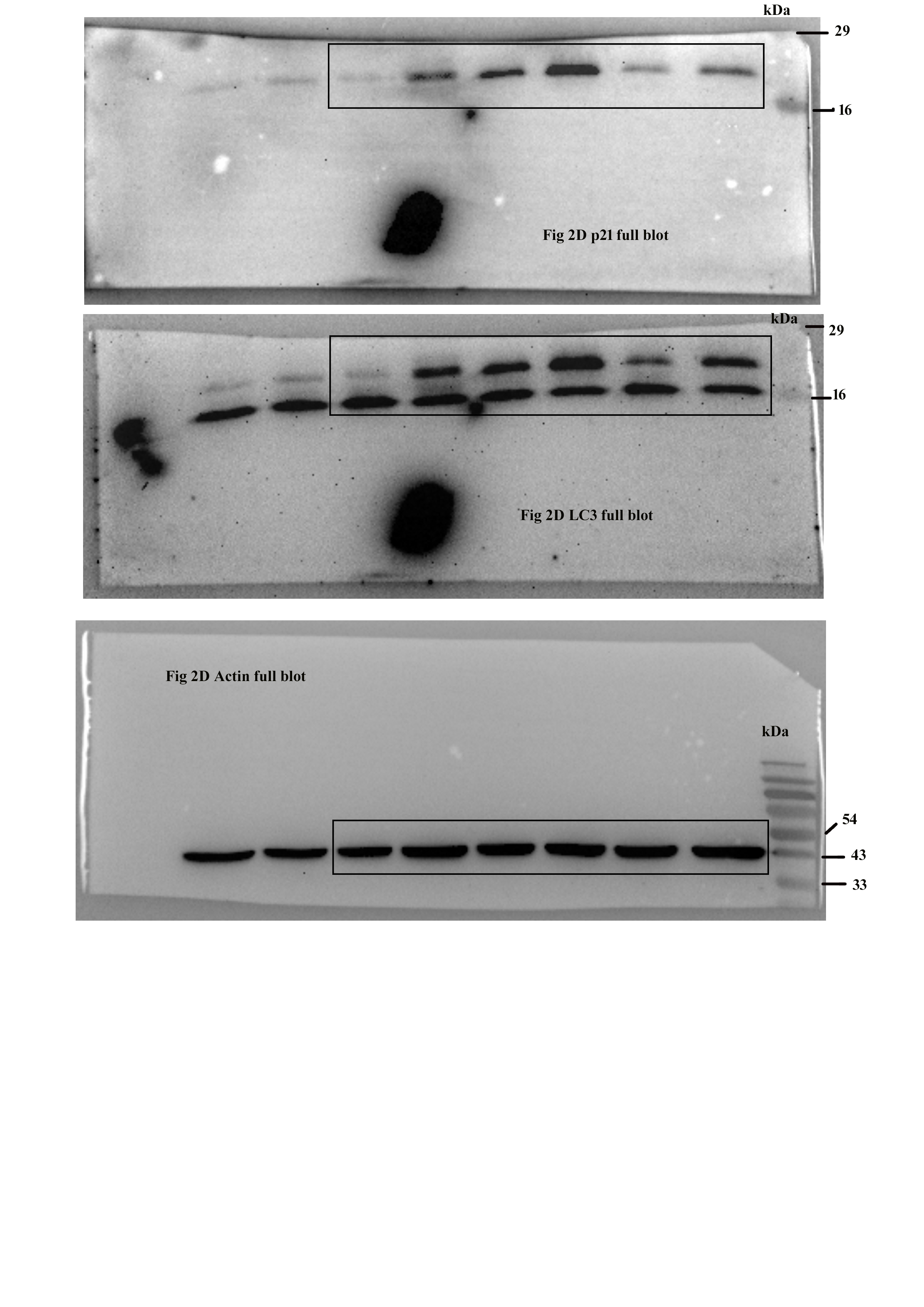

Supplement: Supplementary file 9 — Figure 2D Original Western blots [file 41419_2022_5486_MOESM9_ESM.tif]

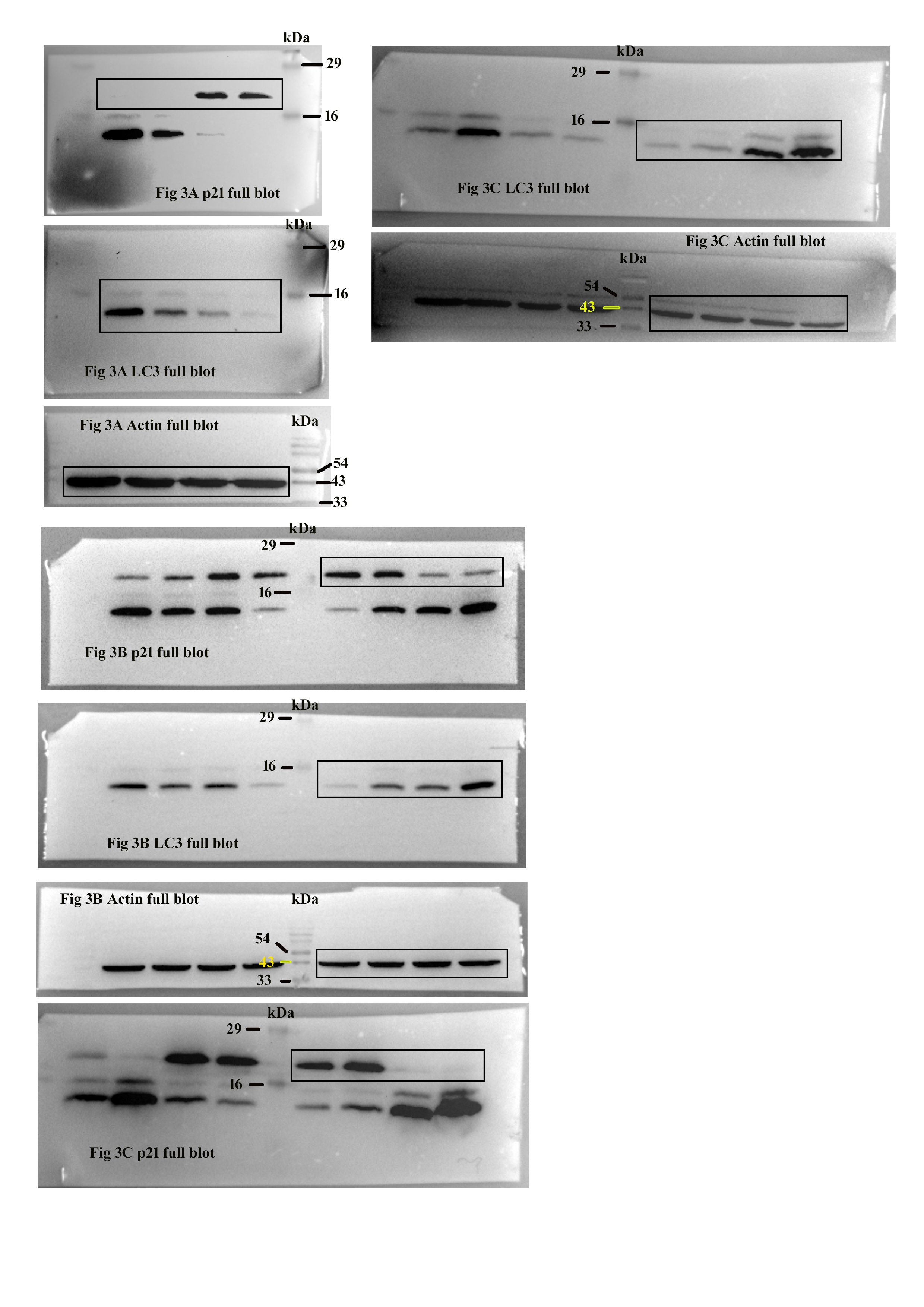

Supplement: Supplementary file 10 — Figure 3 Original Western blots [file 41419_2022_5486_MOESM10_ESM.tif]

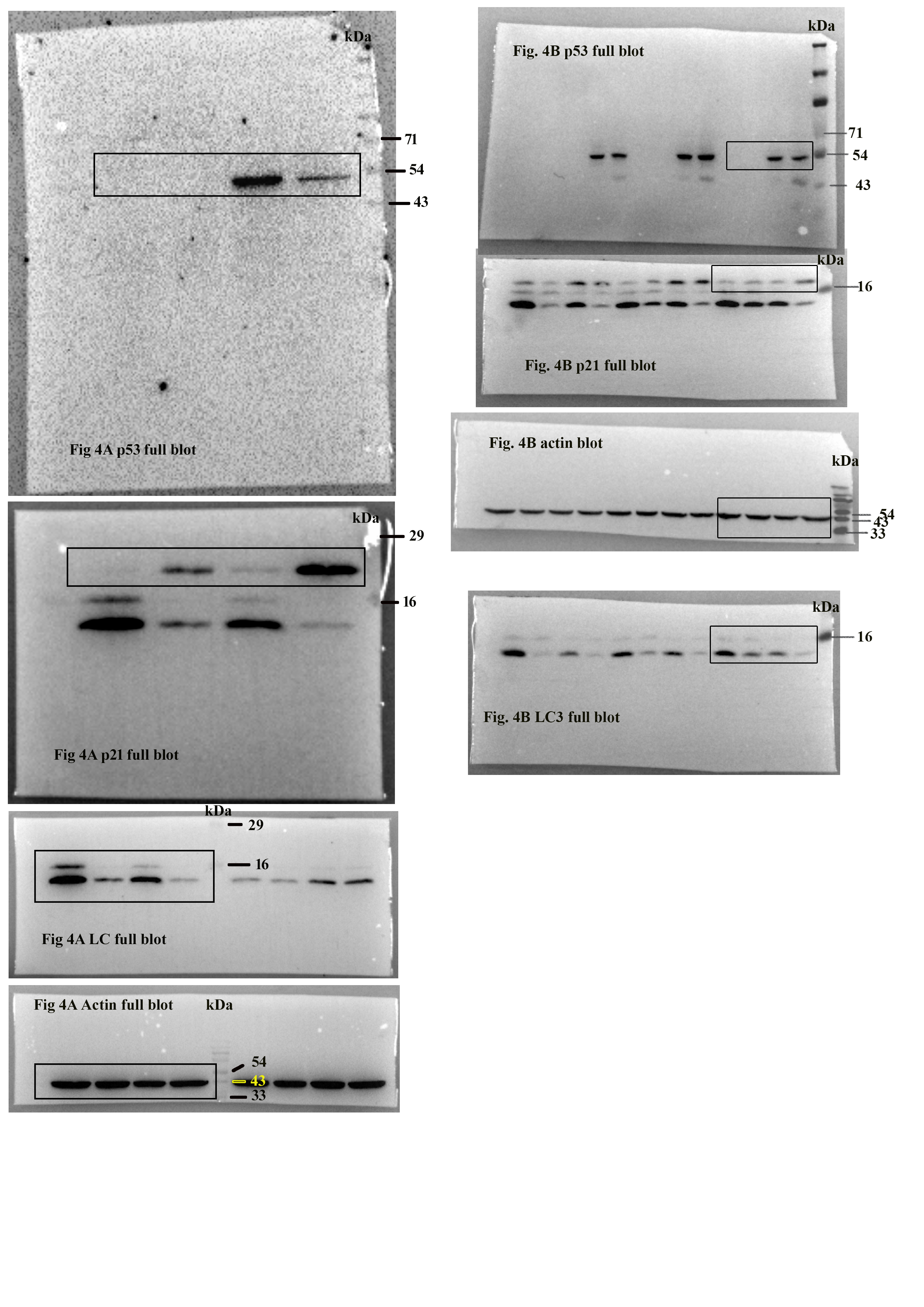

Supplement: Supplementary file 11 — Figure 4 Original Western blots [file 41419_2022_5486_MOESM11_ESM.tif]

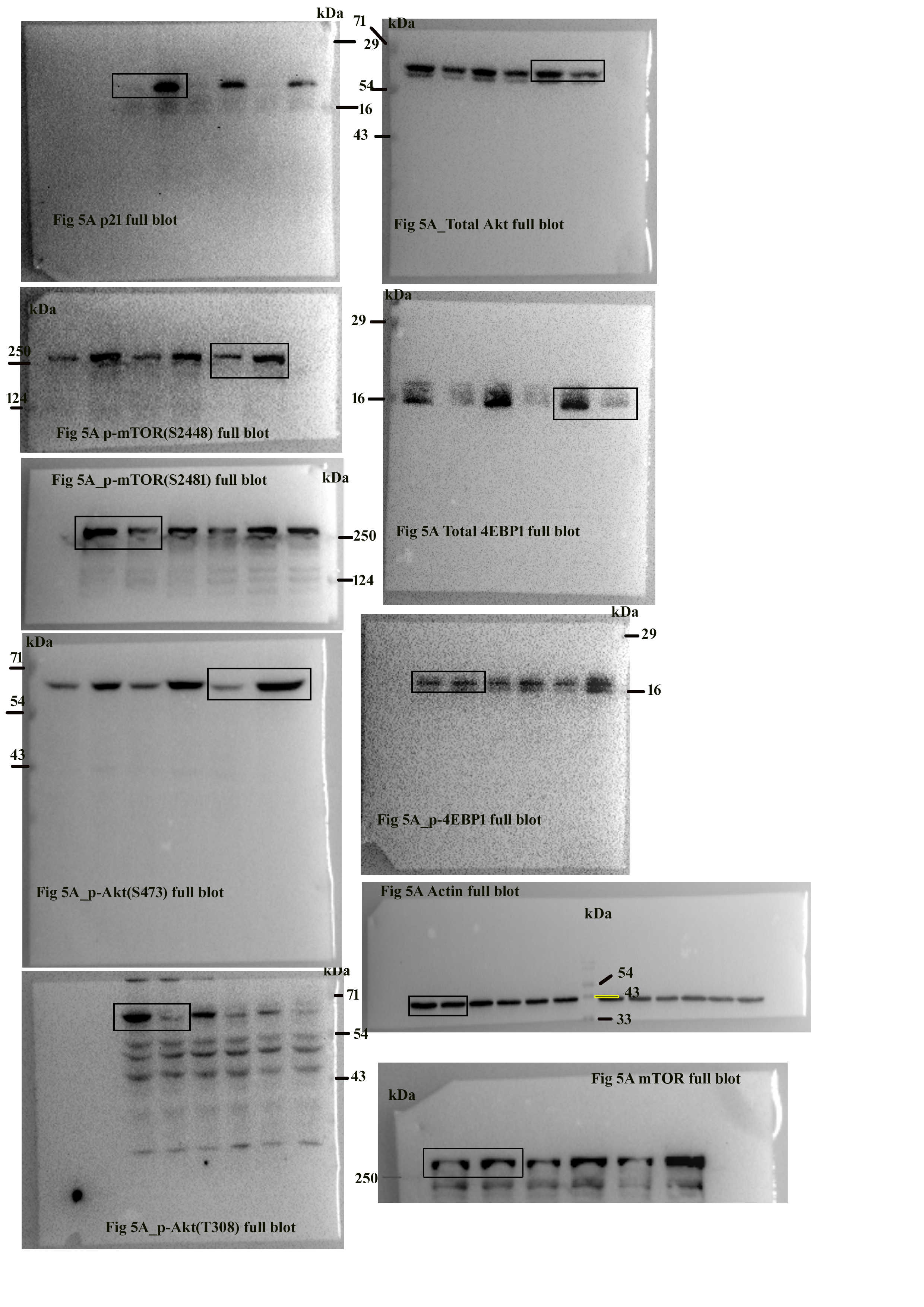

Supplement: Supplementary file 12 — Figure 5A Original Western blots [file 41419_2022_5486_MOESM12_ESM.tif]

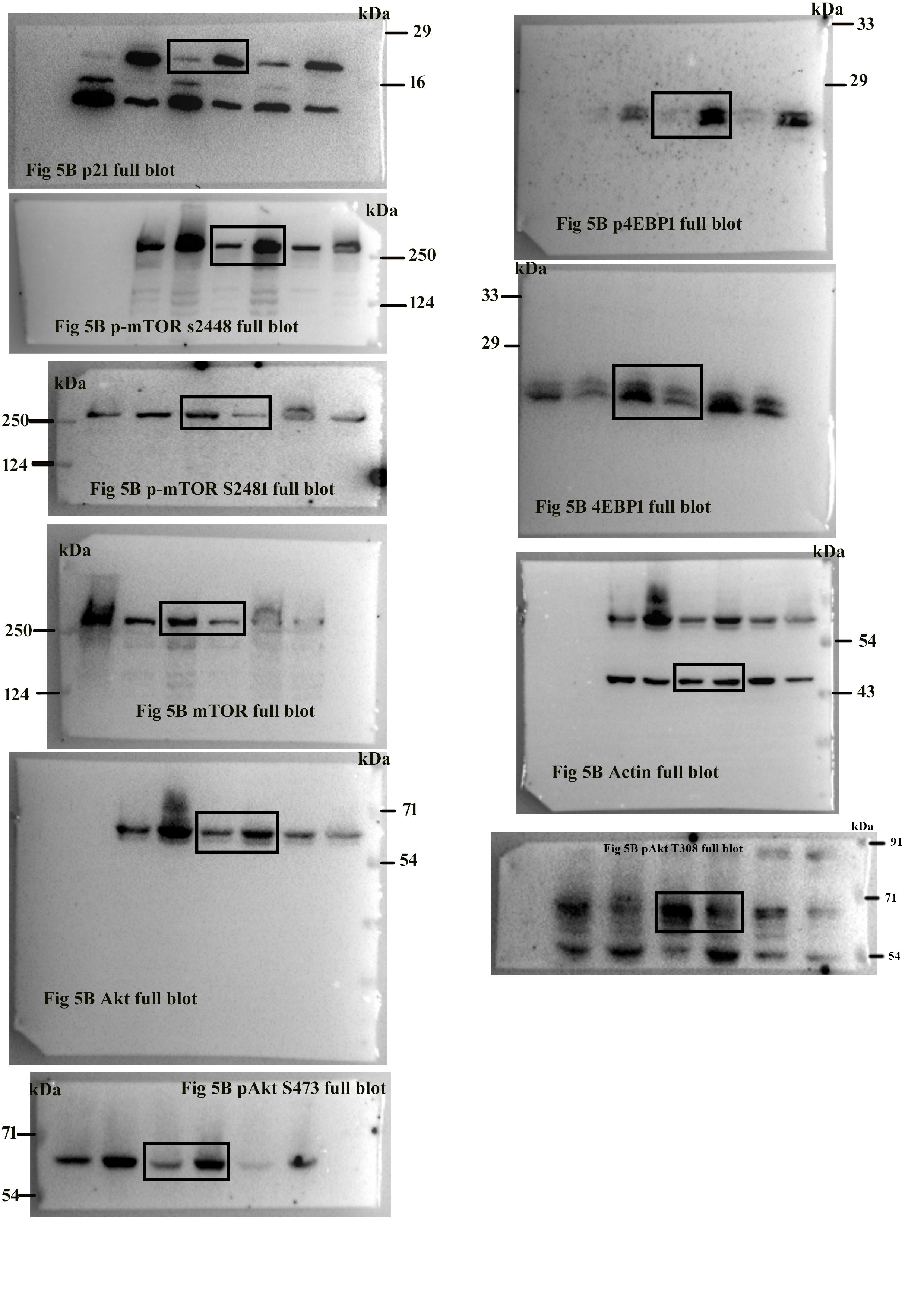

Supplement: Supplementary file 13 — Figure 5B Original Western blots [file 41419_2022_5486_MOESM13_ESM.tif]

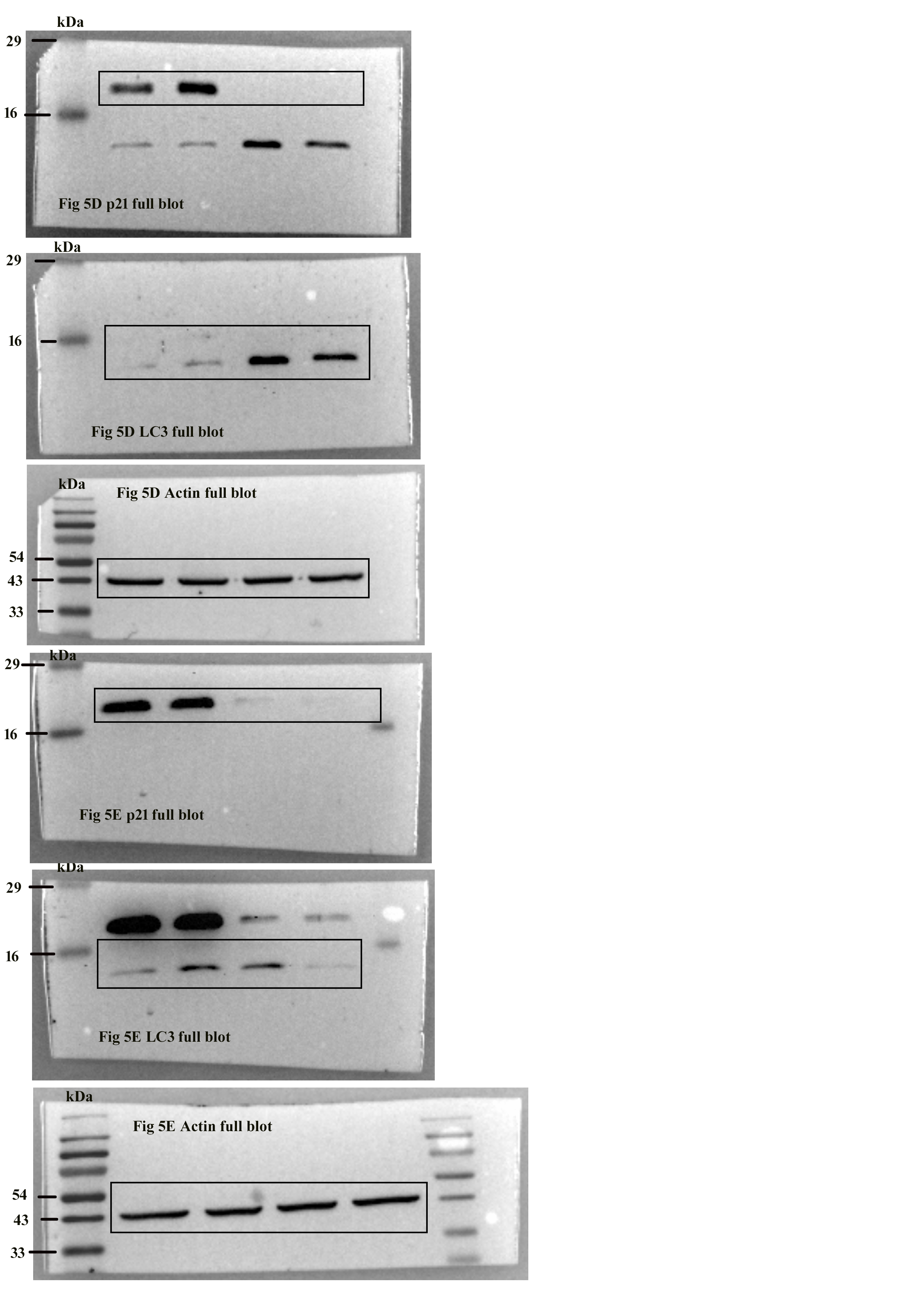

Supplement: Supplementary file 14 — Figure 5D and E Original Western blots [file 41419_2022_5486_MOESM14_ESM.tif]

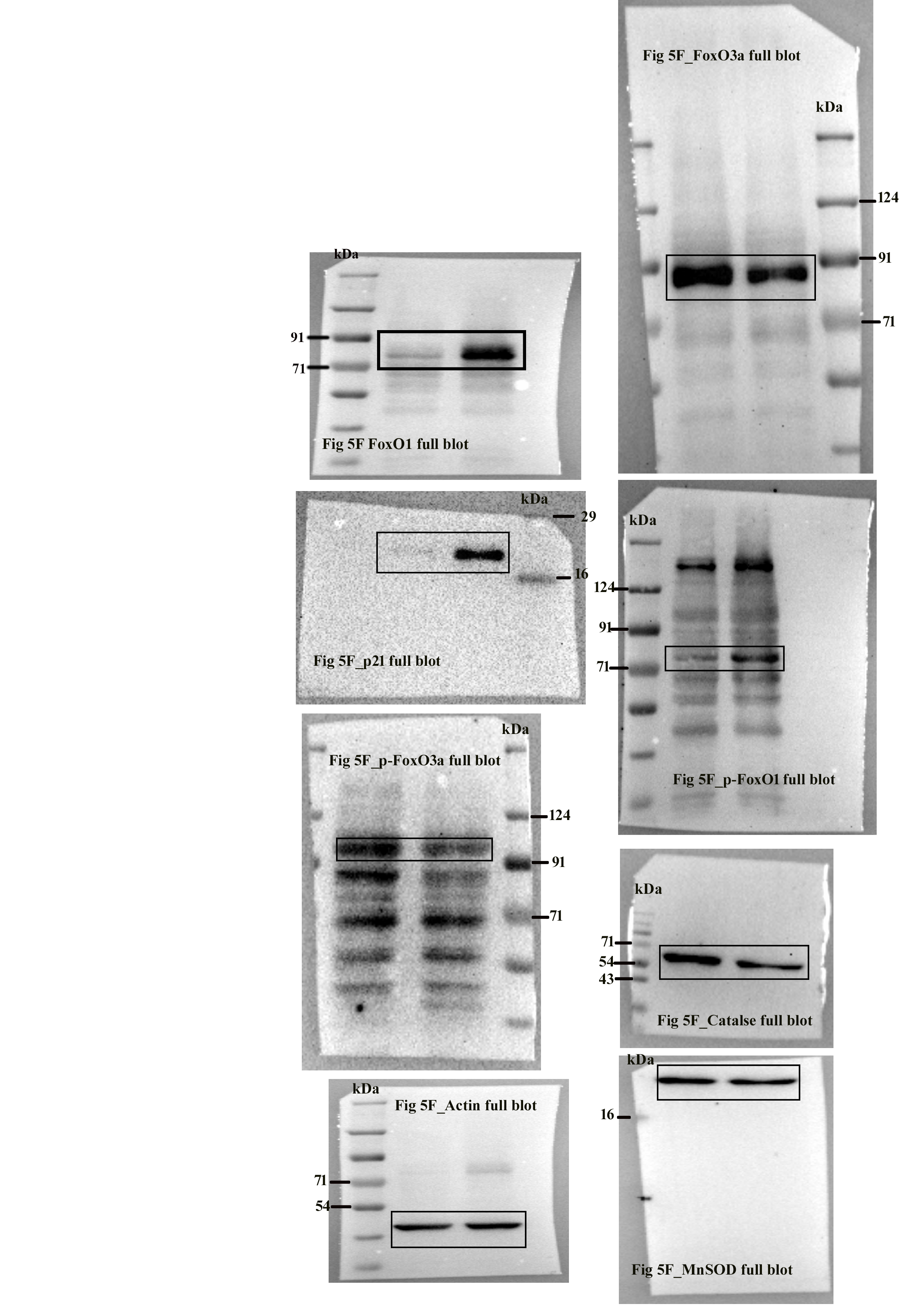

Supplement: Supplementary file 15 — Figure 5F Original Western blots [file 41419_2022_5486_MOESM15_ESM.tif]

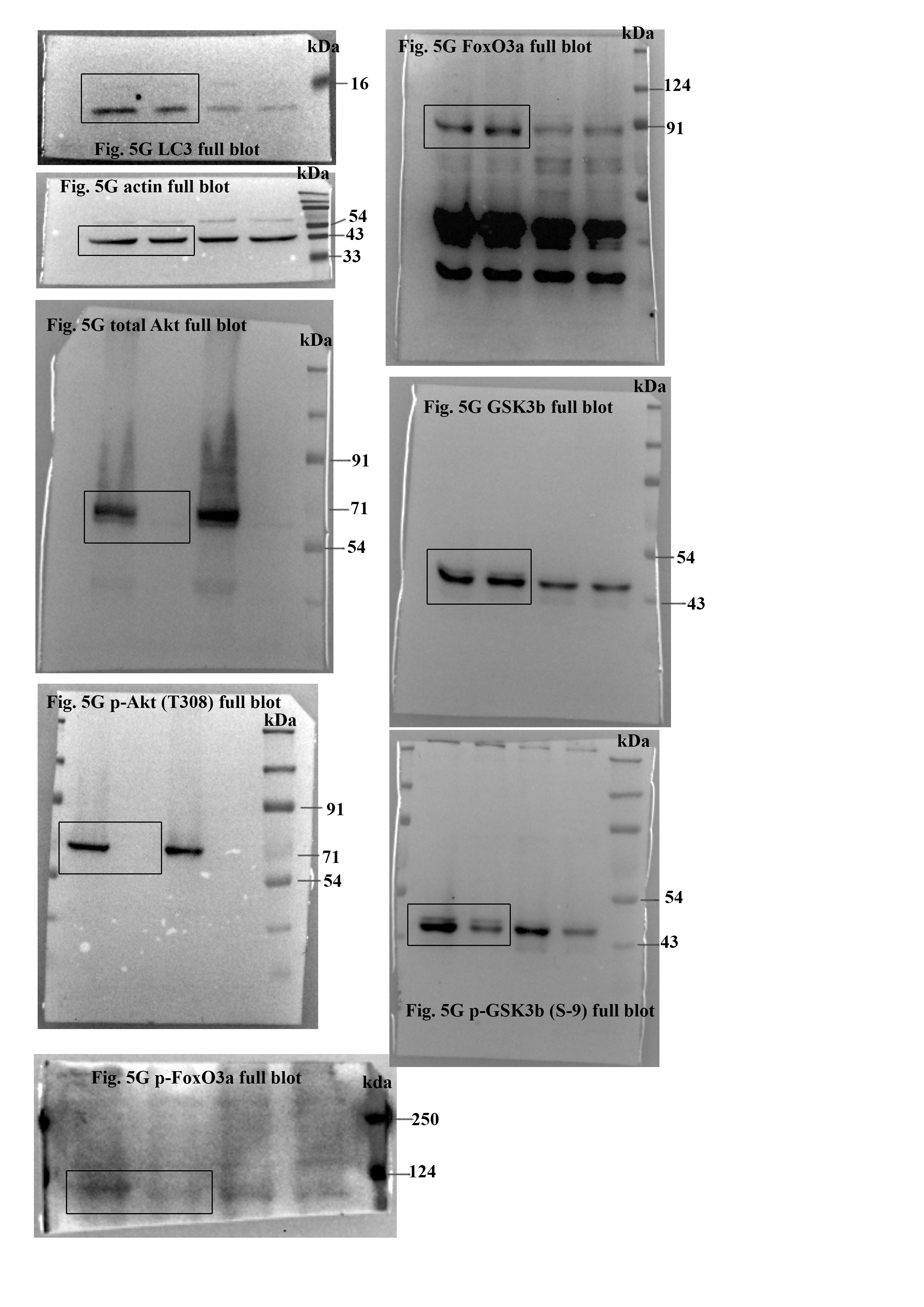

Supplement: Supplementary file 16 — Figure 5G Original Western blots [file 41419_2022_5486_MOESM16_ESM.tif]

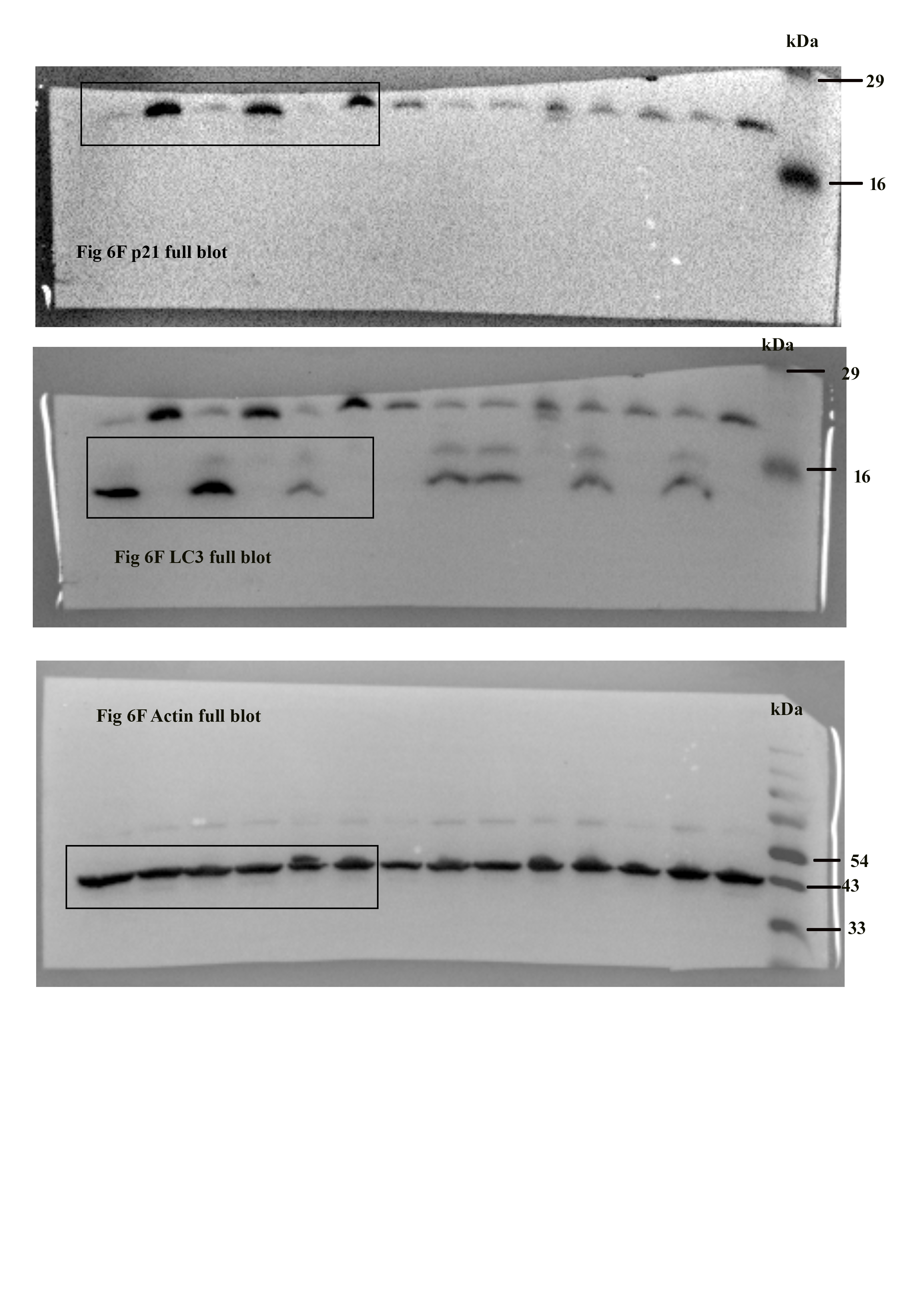

Supplement: Supplementary file 17 — Figure 6F Original Western blots [file 41419_2022_5486_MOESM17_ESM.tif]
